# Fever and health-seeking behaviour among migrants living along the Thai-Myanmar border: a mixed-methods study

**DOI:** 10.1186/s12879-023-08482-8

**Published:** 2023-07-31

**Authors:** Napat Khirikoekkong, Supa-at Asarath, Mayreerat Munruchaitrakun, Naw Blay, Naomi Waithira, Phaik Yeong Cheah, François Nosten, Yoel Lubell, Jordi Landier, Thomas Althaus

**Affiliations:** 1 Mahidol Oxford Tropical Medicine Research Unit, Faculty of Tropical Medicine, Mahidol University, Bangkok, Thailand; 2 Shoklo Malaria Research Unit, Faculty of Tropical Medicine, Mahidol University, Mae Sot, Tak Thailand; 3 Centre for Tropical Medicine and Global Health, Nuffield Department of Clinical Medicine, University of Oxford, Oxford, UK; 4 Institut de Recherche pour le Développement (IRD), Aix Marseille Univ, INSERM, SESSTIM, Aix Marseille Institute of Public Health, ISSPAM, Marseille, France

**Keywords:** Health-seeking behaviour, Fever conception, Febrile illness, Migrants, Thai-Myanmar border, Humanitarian-based, NGO clinics, Healthcare service, Public health system, Migration health

## Abstract

**Background:**

Fever is a common reason to seek healthcare in Southeast Asia, and the decline of malaria has complexified how is perceived, and what actions are taken towards it. We investigated the concept of fever and the determinants influencing health-seeking behaviours among migrants on the Thai-Myanmar border, where rapid economic development collides with precarious political and socio-economic conditions.

**Methods:**

We implemented a mixed-methods study between August to December 2019. Phase I used a qualitative approach, with in-depth interviews and focus group discussions. Phase II used a quantitative approach with a close-ended questionnaire based on Phase I findings. A conditional inference tree (CIT) model first identified geographic and socio-demographic determinants, which were then tested using a logistic regression model.

**Results:**

Fever corresponded to a high diversity of conceptions, symptoms and believed causes. Self-medication was the commonest behaviour at fever onset. If fever persisted, migrants primarily sought care in humanitarian cost-free clinics (45.5%, 92/202), followed by private clinics (43.1%, 87/202), health posts (36.1%, 73/202), public hospitals (33.7%, 68/202) and primary care units (30, 14.9%). The qualitative analysis identified distance and legal status as key barriers for accessing health care. The quantitative analysis further investigated determinants influencing health-seeking behaviour: living near a town where a cost-free clinic operated was inversely associated with seeking care at health posts (adjusted odds ratio [aOR], 0.40, 95% confidence interval [95% CI] [0.19–0.86]), and public hospital attendance (aOR 0.31, 95% CI [0.14–0.67]). Living further away from the nearest town was associated with health posts attendance (aOR 1.05, 95% CI [1.00–1.10] per 1 km). Having legal status was inversely associated with cost-free clinics attendance (aOR 0.27, 95% CI [0.10–0.71]), and positively associated with private clinic and public hospital attendance (aOR 2.56, 95% CI [1.00–6.54] and 5.15, 95% CI [1.80–14.71], respectively).

**Conclusions:**

Fever conception and believed causes are context-specific and should be investigated prior to any intervention. Distance to care and legal status were key determinants influencing health-seeking behaviour. Current economic upheavals are accelerating the unregulated flow of undocumented migrants from Myanmar to Thailand, warranting further inclusiveness and investments in the public health system.

**Supplementary Information:**

The online version contains supplementary material available at 10.1186/s12879-023-08482-8.

## Background

Fever is one of the most frequent reasons for seeking healthcare in Southeast Asia, particularly at the community-level [1–4]. Its burden is illustrated by repeated bacterial and viral outbreaks, as well as the emergence of severe infections such as melioidosis or multi-drug resistant tuberculosis [5–9], and these are frequently reported amongst migrants, mainly due to limited healthcare access [10–13]. In parallel, the burden of malaria has reduced substantially over past decade, through wide access to point-of-care tests, providing a clear therapeutic guidance [14]. Presently, most febrile patients will have a negative malaria test result, and this trend might affect their a priori choice of health service to seek care with [15–17]. A growing diversification of health services has been reported in Southeast Asia which include public and private hospitals, research institute or humanitarian-based clinics, drug stores, private clinics, as well as itinerant-and-often unqualified health workers. However, the healthcare seeking behaviour is further challenged and complexified by individual preference, such as the importance of traditional medicine in ethnic groups, or self-medication with antibiotics [18–21].

Understanding health-seeking behaviour is at the cornerstone of public health strategies. First, it is necessary to understand how the medical concept of fever translates into the understanding, representations and practices of a given population. Although fever may appear as a very simple concept and is widely used in policy-making strategies, it lacks consensus in its definition, even within the medical community, and often includes a myriad of clinical manifestations [22, 23]. Second, believed and actual causes of fever may differ in space and time: in countries with a marked decline of malaria like in Southeast Asia, malaria persists in being considered the main cause of fever [24]. Third, investigating the journey to care during fever and identifying its determinants are critical for designing relevant strategies addressing public health gaps.

The Thai-Myanmar border is an exemplary setting to study these issues: in 2019, the monthly number of migrants crossing the Thai-Myanmar border was estimated at around 200,000 [25]. These migrants have either been impacted by political instability and/or military conflict, attracted by intensive economic development in special economic zones [26]. Migrants in this area are defined by a high diversity of ethnicities and religions [27, 28], while socio-economic determinants point towards having low education, limited income, being undocumented, as well as drug and alcohol addictions [29].

For the past 3 decades, Thai healthcare system development has been revolving around improving quality of care and increase access to primary healthcare services [30]. However, despite good efforts of Thai government to provide basic healthcare to all migrants and to recognizes the right to health [31] being undocumented and having no legal status are the main problems for an estimated 2.3 million undocumented migrants to gain access to healthcare [32]. In response, the Migrant-Fund (M-Fund) was launched in 2017, to provide affordable healthcare coverage to migrants along the Thai-Myanmar border. The M-Fund offers a non-profit health insurance scheme for this underserved population group, and operates in collaboration with Thai public hospitals affiliated to Ministry of Public Health and with local non-government organizations providing free humanitarian healthcare [21, 33]. In spite of these recent improvements, the determinants influencing the journey to care, although acknowledged to be decisive, remain understudied, especially on the border area between Thailand and Karen state, Myanmar. However, Zhang et al. described the journey to care for any kind of illnesses, while the pattern of health-seeking behaviours is known to vary according to the type of disease [34].

Using a mixed-method approach combining qualitative and quantitative analyses, we aimed to understand the concept of fever and its believed causes amongst migrants alongside the Thai-Myanmar border, and explore the association between migrants’ determinants and health-seeking behaviours.

## Methods

### Study context and setting

This study was conducted in Tak Province, in four districts situated along the river Moei, as natural border between Thailand and Myanmar. The majority of the population, including migrants, are farmers, either owning land for farming, or working for other farmer(s). The Karen state, located opposite to Thailand, is known for its long-ongoing conflicts. Occasionally, the villagers crossed the border in seeking for shelter. However, in time of peace, the people regularly cross back-and-forth for various reasons including seeking for employment, seeking for health care, reach other parts of Thailand for work [33].

### Patient and public involvement

Members of the public got involved in this research at different stages. Firstly, our research questions stemmed from the experience of previous local healthcare programmes in the region, carried out by local humanitarian and research organisations to serve marginalised populations. Understanding their motivations when seeking health care and to understand the roots of healthcare seeking behaviour had appeared as an essential component to improve current health services. Therefore, community representatives, as members of the public, Tak Province Community Ethics Advisory Board (T-CAB) were first involved and were consulted during the protocol design, to ensure its relevancy and utility for the community.

Currently, we have a plan to present the published findings and present the video product of an engagement activity with village health volunteers which used participatory visual method and facilitated discussions to create visual outputs.

### Study design

We followed a mixed-methods design consisting of two distinct phases: We used a qualitative approach in Phase I, and we used findings of Phase I to inform quantitative approach of Phase II, including designing questionnaire survey. Topic guide for in-depth interview (IDI) and focus group discussion (FGD) for Phase I study and questionnaire survey for Phase II were designed and developed specifically for this study with consultation of Community Engagements (CE) team from Shoklo Malaria Research Unit (SMRU), as presented in the Additional file 1. For both Phases, the study team conducted reconnaissance visits to each site to explain and introduce the study to healthcare staff and community representatives. It was necessary to build rapport and consult with community representatives in order to avoid and minimize any burdens for participants.

Phase I data was collected from 14 June to 2 October 2019, and Phase II data was collected from 3 November to 19 December 2019.

**Fig. 1:**
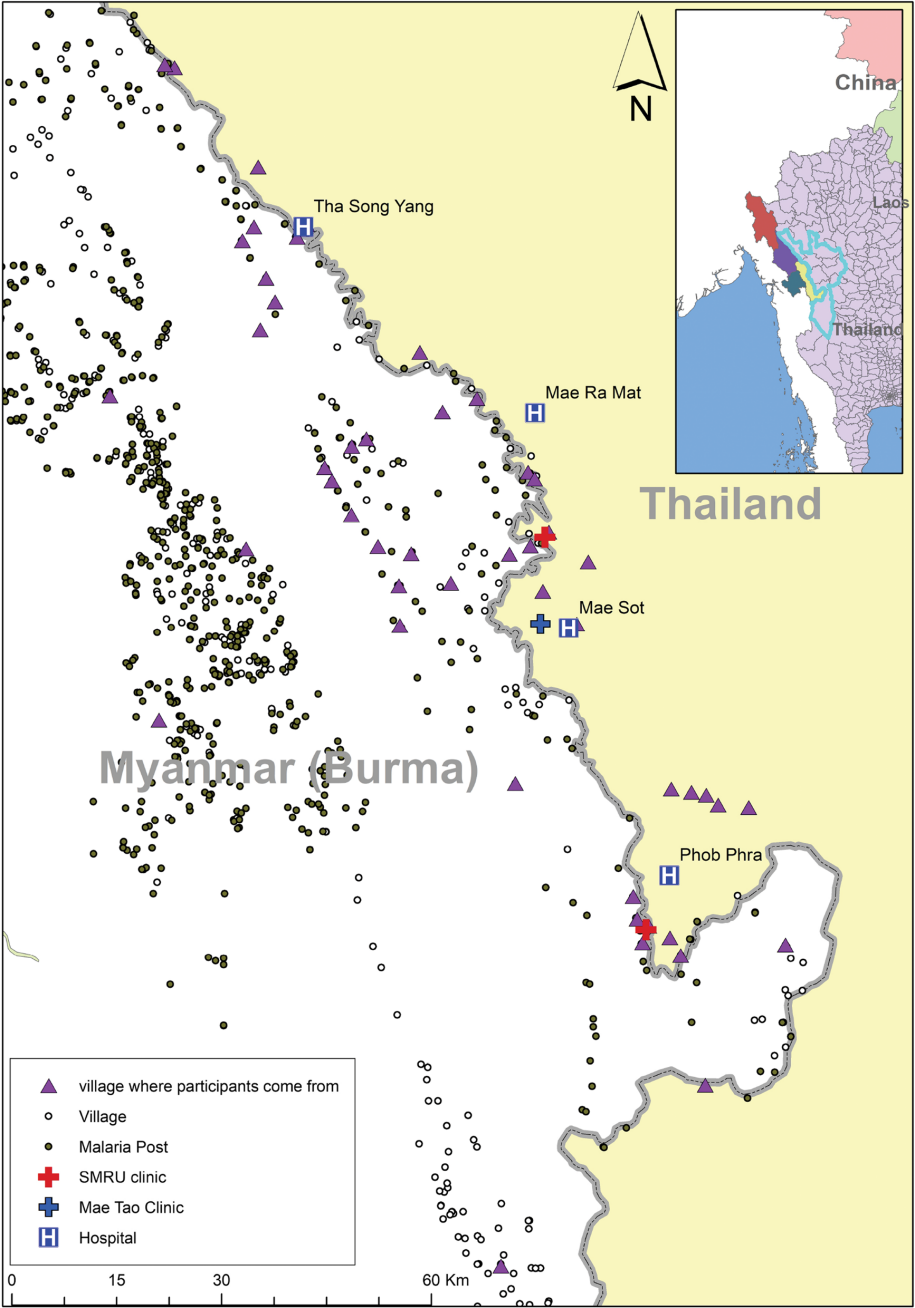
Mapping of study sites and villages. Map generated using ArcGIS pro 2.5

Data collection sites for both Phase I and II were located in four districts of Tak province covering 6,039 km^2^, in Mae Sot (7 sites), Phob Phra (2 sites), Mae Ramat (3 sites) and Tha Song Yang (3 sites). Figure 1 illustrates villages where participants are from when coming to seek healthcare, however, data collection sites were unlisted to protect confidentiality of participants, and as criteria in deidentification. Participants of this study are undocumented migrant workers, and cross border villagers from Myanmar with high mobility and travel around the border area without official permits. Site selection criteria included presence of health centre(s) or clinic(s), proximity to border crossing points, geographical location, and social-culture-economic characteristics, to represent as much as possible the diversity of the population.

### Phase I

Phase I relied on qualitative data collection methods, which included in-depth interviews (IDIs) and focus group discussions (FGDs). The study team purposively enrolled community members aged 21 years old and above, Village Health Volunteers (VHVs), primary care staff, and members of the Tak-Province Community Advisory Board (T-CAB) [35, 36]. Table 1 display detailed breakdown of participants by group and by data collection method.

**Table 1:** Total number of study participants

**Participant group**	**Phase I**			**Phase II**
	**In-depth interviews (in person)**	**Focus group discussion (in group)**	**Participatory visual activity (in person)**	**Electronic questionnaire (in person)**
**Group 1** Community members	24	-	-	202
**Group 2** Village Health Volunteers, primary care staff	9	2	-	-
**Group 3** Key informants, T-CAB^a^ members, PCU^b^ staff	-	1	8	-
**Total**	**33 persons**	**3 groups (15 persons)**	**8 persons**	**202 persons**

^a^*T-CAB* Tak-Province Community Advisory Board

^b^*PCU* Primary care unit

The participants of group 1 were interviewed individually to gain as much diverse data as possible, which contributed to data saturation and confirmation of themes emerged, and to help understand the health-seeking behaviour of migrants/community members in case of acute undifferentiated febrile illness. The insights, and perspective, and experiences of healthcare workers are not only how community members perceived fever and its causes, but also how they chose to act at fever onset. Focus group discussions further strengthened integrity of data and emerged themes across all participant groups. Especially, the narrative from key informants group discussions helped confirming baseline for conception of fever, and for therapeutic itinerary(s). Invited participants had deep understanding of the challenges, limitations, and behaviours of migrants in this area. PCU staff provided insights about the health seeking trend across PCU network, as well as treatment expectations in general.

Potential group 1 participants were approached by study team while they were seeking health care services at the health centres. Group 2 and 3 participants were identified through existing contacts, and snowballing technique was used to recruit participants of the same group. The study team proceeded with participation information session and informed consent process after obtaining an informal agreement from the potential participant. All participants, either as individual or as group for FGD, were reminded that the session will be audio recorded for formal transcribe and analysis. The audio recorded from Phase I are stored in a secure storage system of Mahidol Oxford Tropical Medicine Research Unit (MORU), and on encrypted external hard-disk dedicated to this study. All transcripts will be de-identified.

The majority of interviews and focus group discussions were conducted in Karen and Burmese, while a minority were conducted in Thai. Interview sessions lasted between 45 min to 1 h, and focus group discussions lasted between 1 h to 1.5 h. Interviewers for Phase I are native Karen/Burmese speakers with deep understanding of research setting and the cultural context (co-authors NK, MM and NB), interviews in Thai were led by SA.

To complement Phase I, a participatory visual method (PVM) workshop was carried out with eight people who voluntarily serve as village health volunteers (VHVs). The PVM participants were not included as study participants. The workshop employed participatory tools and focused on engaging and exploring the ‘conception of fever, believed causes and symptoms, and the corresponding journey to care’ from invited participants. Activities in the workshop emphasised on group discussion and production of drawing to illustrate the visual conceptions, rather than solely use verbal communication. Karen language was preferred by participants to use in the workshop, mainly led by NK. Visual outputs i.e., co-created drawings from facilitated discussions, had contributed to a video output, produced by SA, and shown to participants as part of the engagement activity [37].

Phase I data analysis relied on a thematic analysis, to formulate and derive patterns of health-seeking behaviour in case of fever [38]. Data collection for Phase I stopped once data saturation was reached and no new themes emerged.

NVivo data management software (QSR International, Melbourne, VC, Australia) was used to code de-identified transcripts, and to organize and manage qualitative data of Phase I.

### Phase II

In order to identify potential associations between migrant’s determinants and health-seeking behaviour in case of fever, we collected quantitative data using a questionnaire survey based on findings from Phase I i.e., IDI and FGD. We developed an online electronic questionnaire using OpenDataKit (ODK) software, and collected all responses using ODK Collect application on tablets. The study team (MM and NB) were trained by NK and SA on electronic data collection with a pilot test to identify any ambiguities.

Phase 2 participants were community members of Karen and Burmese ethnicity, aged 21 years old and above, and living in the four border districts or living in Karen State and crossing the border regularly. Establishing a sampling frame in the context of unregistered and mobile population is challenging and cannot rely on any national demographic census [39]: therefore, enrolment was carried out in field sites where migrants are commonly live and work [10, 40, 41]. Study sites consisted in border crossing points, communities located within walking distance from the border, and health centres or clinics where potential participant usually attend. Participants were invited to join the survey based on a convenient sampling method in all sites. The study team stationed about 5–6 h during a day in a given site, approaching people crossing the border or accompanying patients to explain the study, and included volunteers. Each questionnaire took approximately 20 min to complete, after which the study team approached the next volunteer.

In the collection process, study team member read out each question in Karen or in Burmese and enter responses into the application. The study team checked the collected forms on the tablets on a weekly basis, to correct any language issues, including spellings and translations before finalising the forms and sending them to a secured central server for storage. If responses were too long to enter into the tablets in a timely manner, study teams took note of responses on separate paper, or onto a printed version of questionnaire. Participants in Phase II provided written informed consent individually, and well as tick confirmation boxes at the start of questionnaire. Phase II online questionnaire data is stored in MORU secure and authorized access only server.

For both phase I and II, during informed consent process, participants were informed that they could withdraw from the study at any moment they wish, and there would be no consequences or affect to healthcare at the health centres. This study excluded anyone under 21 years old from enrolment.

### Sample size

For Phase I, the data collection was on-going until the data was saturated and there were no new themes emerged.

For Phase II, we assumed that approximately 50% of our Phase II participants would use cost-free, humanitarian-based health services (i.e., SMRU clinics and Mae Tao Clinic). To obtain a 95% confidence interval of 15% (precision of ± 7.5%), a minimum of 171 participants was required, rounded at 200. Since, participants enrolled in Phase II are those that come to cost-free, humanitarian-based health services. There may be presence of the patient that came to the clinic for treatment or care.

### Primary outcome

Our primary outcome was the attendance at each of the five health services present on the Thai-Myanmar border in case of fever persistence: health posts, private clinics, primary care units (PCU), cost-free clinics or hospitals.

### Secondary outcome

The secondary outcome was the health-seeking behaviour at fever onset, categorised in four groups: “Wait & See”; self-medication; seek health in a regulated or in an unregulated health facility.

### Statistical analysis

Descriptive statistics of continuous variables with normal distribution used means and standard deviation (SD) and medians with interquartile range (IQR) for non-normally distributed variables.

Socio-demographic determinants included were age, sex, marital status, ethnicity, religion, household size, education level, main activity, main and secondary source of income and legal status. Geographical determinants were included in regards to health service accessibility: country of residence (Thailand/Myanmar), distance to the nearest town (in Myanmar or in Thailand) equipped with a public hospital, and whether the nearest town included a cost-free clinic and a public hospital or a public hospital only.

We used conditional inference trees (CIT) to explore the association between the determinants associated with each outcome, testing geographic and socio-demographic determinants independently [42, 43]. Conditional inference tree (CIT) is a method similar to classification and regression tree (CART) limiting bias in variable selection [44, 45]. We used the following CIT parameters in R-package {party}: cquad test, distribution estimation by Monte Carlo method with 5,000 replications, 0.8 = 1 – α as splitting criteria value of statistic test, and a 10-individual minimum node sample size.

Second, univariate and multivariate logistic regression analyses were conducted, and crude and adjusted odds ratio (aOR) with 95% confidence interval (95% CI) were calculated using the R-package {mgcv} [46]. For the secondary outcome, with four categories, multinomial regression was not conducted as no determinants could be identified by the CIT approach. Statistical analysis was performed using STATA version 15 (College Station, Texas, USA) and R 4.0 software.

## Results

We present the results by themes identified from Phase I qualitative data; following by Phase II quantitative analysis, which completes Phase I data – for each theme, qualitative data are followed by quantitative data.

Our results begin with how the population in our study context express out verbally when describe fever and how they understand fever. Then, explore into the symptoms of fever and what are believed causes, and display an illustration of strategies taken in the health-seeking behaviour at fever onset, and when the fever persists. We also provide illustrative quotes – anonymised, noting participant number, and indicate those from individual in-depth interview and group discussion, and also participant group where relevant.

### Concept of fever

From Phase I, the data informed that term “fever” was mainly referred to as “*Ta Nya Ghoe*” in local Karen language, which can also mean “malaria” because malaria was prevalent and had been the common issue in the area for decades. “Fever” was also used to describe a high temperature as an early symptom of any infection. Another description of the term “fever” referred to hot body, warm skin and feeling discomfort. Contextually, the population in the area used the perceived symptoms related to fever when referring to the term “fever”. Hence, there were no direct translation to the word itself for some participants. The diversity of terms referring to “fever” on the Thai-Myanmar border is illustrated in Additional file 2, Fig. S9.

The quantitative analysis (phase II) confirmed the diversity of terms referring to “fever”, with 39 out of 202 (19.3%) participants citing two to three different terms, “Ta Nya Ghoe”, “Malaria”, “Oh Ta Sut”, “Nay Ma Kao Pu” and “Dengue”. The majority of our participants did not use the term “fever” (109/202, 54.0%), and rather described a physical complaint such as “being sick” (69/202, 34.2%) or symptoms such as muscle pain or cough (17/202, 8.4%). We also found significant differences in the terminology of fever between Karen and non-Karen ethnicities (see Additional file 3, Table S1).

*037: “most of them when they get fever, they understand that they have got some kind of bug [virus/parasite]. They understand the most about malaria, as for other diseases, they do not understand much. Mostly because they are told [health knowledge] about malaria for many times, for many times and when they get fever, they would say that it’s Ta Nya Ghoe [malaria].”* FGD, cross border health worker

### Symptoms & believed causes

Symptoms participants described as relating to fever included low or high temperature, shivering along with body aches, headache, runny nose and fatigue. The believed causes and symptoms of fever broadly varied from one participant to another, as illustrated in the following quotes.

*007: “Fever is when we feel that our body is warm, it’s hot in our skin and having headache, also body pain, and do not feel comfortable when our body temperature is high.”* – IDI, female participant

*009: “when having fever/feeling feverish, it’s because of working [outdoor] in the rain and become feverish. Having fever with headache, sometimes with pain on arms and legs. Some with pounding heart, shaking body, cold hands and cold feet.”* – IDI, female participant

Quantitative analysis (phase II) confirmed that most participants associated fever with body ache (151/202, 74.8%), or hot body (142/202, 70.3%). Fever was associated with a broad spectrum of localising symptoms, such as headache (148/202, 73.3%), respiratory (48/202, 23.8%), digestive or urinary complaints (22/202, 10.9%).

Fever was commonly believed to be caused by seasonal change and being exposed to hot or cold weathers. Some shared that having been in the rain, taking shower late in the evening or getting mosquito bites could also cause fever. Key informants and well-respected elders in villages cited spirits as a common cause of fever as well. One of the reasons commonly cited during FGD explaining the importance of traditional belief was the lack of health education and culture inherited from participant’s elders and ancestors.

*037: “Because they do not have health knowledge, when they have fever, they believe that they are being punished by spirits. For the ones living near the city, they know that they have to get their fingers pricked but they do not neglect their traditional belief. They follow both things [modern treatment and traditional/spiritual treatment]”* – Group discussion, cross border HW

Quantitative analysis (phase II) found environmental factors as the main believed cause of fever, including walking in the rain, taking a shower late in the evening or hard working (115/202, 56.9%). A pathogen such as dengue, chikungunya or influenza virus and a mosquito bite were believed to cause fever among 20% (40/202) of participants. A respiratory, digestive or urinary tract symptom was believed to cause fever among remaining participants (35/202, 17.3%). Spirits were not cited as a believed cause of fever in the quantitative analysis.

### Health-seeking behaviour at fever onset

**Fig. 2:**
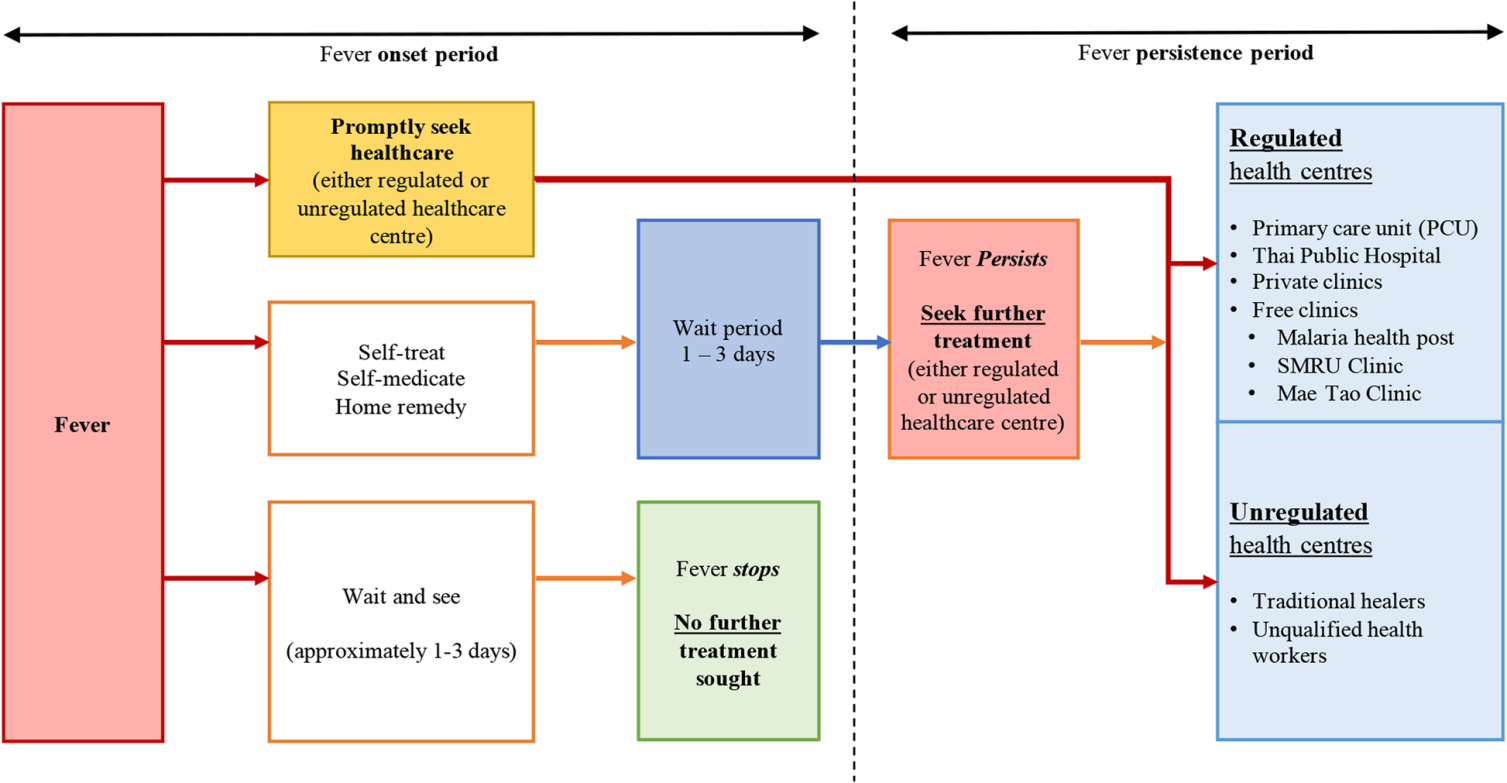
Health-seeking strategies of migrants on the Thai-Myanmar border by period of fever occurrence

The strategy for seeking healthcare was composed of two distinct periods in the occurrence of fever: what migrants do at fever onset; and what else do they in case of fever persistence? Figure 2 illustrates the steps undertaken.

At fever onset, three different strategies were identified in the qualitative analysis: 1) Promptly seek healthcare, either at regulated or unregulated health centre; 2) Self-treatment then wait to see if fever persists or stops; 3) “Wait and See”, no further treatment sought if fever stops, However, in case fever persists, then seek further treatment at either regulated or unregulated health centre.

Self-treatment was a common behaviour at fever onset, and included taking left-over medicines such as paracetamol, or buying poly-pharmaceutical packs from a nearby grocery shop, or taking herbal medicine. The poly-pharmaceutical packs are seen as a quick-fix set of medications generally include steroids and/or antibiotics. Self-treatment also include body rubbing with a soaked towel or taking a shower in hope to cool down the body temperature. Finally, a few participants declared seeking care nearby their home, and cited health post, unqualified health workers, while some would seek care from a traditional healer, particularly when living in remote area.

*013: “Sometimes when have high fever, I rub [my body] with a soaked towel and it cooled down. Sometimes go to buy medicines also. Sometimes… I drink [herbal] water and sometimes I drink raw betel leaf juice that also helps.”* – IDI, male participant

For all these different behaviours, the waiting period before seeking higher levels of care was usually between 1 to 3 days, in case of fever persistence.

*005: “After 2 days and I came here [MKT clinic]. If got better then [I] don’t come, only if not better then I come [to MKT clinic].”* – IDI, male participant

The qualitative analysis (phase I) highlighted factors influencing participants’ rationale for seeking care at fever onset: living far away from a healthcare facility was one of the most frequent reasons for participants to stay home wait and see, or self-medicate. Participants cited long traveling hours with high costs, as well as lack of transports to reach higher levels of health centres.

*005: “[I] live far away from the clinic and [I] could not come. For that reason, I gave him [child] medicines based on my own understanding [on medicines]. Only if he did not feel better, we would come here [MKT clinic]. If the medicines he took made him better, we would not come here. Because [we live] far away from clinic, for that reason we don’t come. But, if he is not better, we would come [to clinic] with bicycle, we would rent other people’s motorbike, or we would come on foot early in the morning.”* – IDI, male participant

Nevertheless, being ill and absent from work resulted in loss of income, especially for men, who are head of household, and responsible to provide financial security to their family. Phase I indicated that adult males chose to self-treat and buy sets of polypharmacy for quick-fix solution from nearby grocery shops, while several preferred injections by unqualified health workers. Some male participants sought care from private clinics even if the cost is high, because of a perceived faster recovery than going to other facilities, such as public hospitals or health posts. Fast recovery was critical according to the qualitative analysis, especially because being absent from work means losing jobs and loss of income.

*035: “A lot of people selling medicines and a lot of people buying taking and injecting without understanding. They do not go to hospital/clinic.”* – FGD, cross border HW

*011: “Yes, [name of private] clinic. When I get treatment there, I have to pay but I get well, I recover fast, too. The doctor is also nice. There are quite a lot of people at the [public] hospital. If [I am] not severe, I won’t go to [public] hospital. If I want to get and injection, I go to [private clinic]. Just one injection and I get cured.”* – IDI, male participant

The quantitative analysis (phase II) was consistent with Phase I findings regarding participants’ strategy at fever onset: self-medication was the commonest strategy (123/202, 60.9%), mainly through a grocery shop (71/123, 57.7%), and left-over drug from home (52/123, 42.3%). The second most frequent strategy was to attend regulated healthcare such as a health post, cost-free clinic, PCU or hospital (31/202, 15.3%), followed by unregulated healthcare (29/202, 14.4%), such as traditional healers (22/29, 75.9%) or *Mor kapao* (unqualified health worker, 7/29, 24.1%). The least chosen strategy at onset fever was to “Wait & See” whether fever could resolve by itself (17/202, 8.4%). Of 202 participants, 69% (*n* = 140) reported waiting for 1 day before seeking care, 22% (*n* = 45) reported 2 days, and 8% (*n* = 17) reported 3 or 4 days.

Among participants choosing self-medication at fever onset, exclusive paracetamol intake was the most preferred option (126/202, 62.4%), followed by herbal medicine and poly-pharmaceutical packs which is referred to as “*Ya chood”* (equally chosen in 29/202, 14.4% each). Taking an antibiotic or an antimalarial was the least chosen option at fever onset (9/202, 4.5% and 2/202, 1.0%, respectively), while the rest of our participants did not know what drug they were taking (3.3%). None of the geographical, demographic and socio-economic determinants were found to be associated with health seeking behaviour at fever onset using the CIT model. The details of all participants’ determinants by health seeking behaviour at fever onset are detailed in the Additional file 3, Tables S2 and S3, which presented options of health services originate from the qualitative component of the study (phase I).

### Health-seeking behaviour in case of fever persistence

The qualitative analysis (phase I) revealed that only SMRU and Mae Tao clinics provided free health care for both documented and undocumented migrants on the Thai-Myanmar border (Table 2). Apart from cost-free services, these clinics offer a broad range of diagnostic and therapeutic tools for febrile patients. Undocumented migrants face challenges when accessing care at hospitals and PCUs as they would be charged, and such cost can be doubled when attending a private clinic. Most undocumented migrants in this area are earning under about THB120-150 per day (or about USD3), and face unstable employment: as a result, affording health services at facilities which are not free of charge is a challenge.

**Table 2:** Health services available in case of fever persistence on the Thai-Myanmar border

**Health service provider**	**Services provided for febrile patients**	**Costs in relation to legal status when accessing care**		**Language**
		Undocumented	Documented or with health card/ health insurance	
Health post	Community care	Free	Free	Burmese, Karen
Private clinic	Primary care	$$$	$$$	Thai and Translator
Primary care unit (PCU)	Primary care	$$	Covered	Thai and some translation service
SMRU & MTC clinic ^a^cost-free clinics	Secondary-level care	Free	Free	Burmese, Karen
Thai public hospital	Secondary-level care	$$	Covered	Thai and some translation service

Community care: Malaria test; Artemisinin-based combined therapy (ACT); Antipyretic (e.g., paracetamol); manage by community health worker (CHW)

Primary care: PCUs provide oral and intra-venous antibiotics; Frontline disease control and screening unit; Promote and follow-up on health and hygiene; Provide antipyretic (e.g., paracetamol); Rural PCUs only offer consultation without treatment; Management by medical doctor

Private clinics: Provide oral and intra-venous antibiotics, and common antipyretic (e.g., paracetamol); Offer health consultation and treatment; Management by Ministry of Public Health

Secondary-level care:

- Shoklo Malaria Research Unit (SMRU) clinics and Mae Tao Clinic (MTC) – Malaria test; ACT; Dengue and chikungunya virus rapid test; Oral and intra-venous antibiotics; Antipyretic (e.g., paracetamol); Maternal & Child health; Vaccination; Inpatient ward; Management by medical doctor research institute

- Thai Public hospital – Malaria test; ACT; Blood culture; Oral and intra-venous antibiotics; Antipyretic (e.g., paracetamol); Maternal & Child health; Vaccination; Inpatient ward; manage by Ministry of Public Health

$$ = costs not exceed 300 Thai Baht; $$$ = costs exceeding 300 Thai Baht - based on minimum daily wage

^a^Cost-free clinics provide free-of-charge health care services to all migrants and cross-border population living along Thai-Myanmar border area

Indirect cost due to distance to care was also highlighted in the qualitative analysis, representing an additional burden, especially for undocumented migrants. However, for some, they were able to afford hospital insurance, which reduces the cost of future care.

*007: “Before, when I didn’t have any documents, I paid quite a lot, for example when I came to [PCU for] antenatal for the first time, I paid 300 baht (USD6-7), then the second time around 100 baht (USD3), because a doctor told me to get a card at [district] hospital. The hospital issued me a card, then I paid 30 baht (USD1) for medicine each time.”* – IDI, female participant

Both documented and undocumented migrants faced several challenges in accessing healthcare, but the qualitative analysis revealed the importance of legal status as a key factor, especially Thai health services, such as PCUs or hospitals. Health options available to migrants without legal status were reduced to health posts and cost-free clinics. Most migrants living in rural areas described additional difficulties because of transportation, as reaching an official healthcare facility may require several hours and different modes of transports. We identified a double burden when traveling to healthcare facilities – even to free-of-charge facilities: being arrested, fined, and deported by absence of legal status; and travel costs because of living in remote area. The qualitative analysis also revealed communication issues when attending Thai health services, since most migrants only speak Karen and/or Burmese. On the contrary, health posts and cost-free clinic staff belong to the local community.

*040: “they come here; they don’t want to go to the Thais’. They have a weakness on not being able to speak the language [Thai], for some and for others its financial issues.”* – IDI, female HW

Qualitative analysis (phase I) also included health workers from cost-free clinics, Thai PCUs, and CHWs living on both side of the Thai-Myanmar border area. Barriers to care related to legal status were frequently mentioned, particularly when referring a patient to a high facility level, such as hospital. The quote below summarises the challenges encountered by cross border health workers.

*035: “For us to refer patients, we also have difficulties. There are three difficulties. First, there is no transportation fees to travel. Second, to refer to Thai hospital, they do not understand the language. Third, they do not have any kind of documents/IDs. So, one is financial issue, one is language issue, and another one is the absence of documents and guarantee [for safety], which is a major problem.” –* Group discussion, male cross border HW

In quantitative analyse (phase II), there were 22 (out of 202 participants (10.9%) who responded "No" to all options in case of fever persistence, while 74 (36.6%) reported access to one type of health facility, 58 (28.7%) reported two, 34 (16.8%) reported three, 12 (5.9%) reported four and two (0.5%) reported access to five types of health facilities.

The facility most attended in case of fever persistence was cost-free clinics by 45.5%, 95% CI (38.5%-52.7%), followed by private clinics (43.1%, 95% CI [36.1%-50.2%]), health posts (36.1%, 95% [CI 29.5%-43.2%]); public hospitals (33.7%, 95% CI [27.2%-40.6%]), and PCUs (14.9%, 95% CI [10.2%-20.5%]). The geographical distribution of attendance to each type of health service in case of fever persistence is presented in the Additional file 2, Fig. S1.

The quantitative analysis (phase II) investigated all demographic, geographical and socio-economic determinants with regards to health seeking behaviour in case of fever persistence (Additional file 3, Table S3). Determinants selected by the CIT model as significantly associated with health services attended in case of fever persistence are presented in Table 3 below. If the town nearest to where they lived had a cost-free clinic, participants were less likely to attend health posts and public hospitals (aOR 0.40, 95% CI [0.19–0.86] and aOR 0.31, 95% CI [0.14–0.67], respectively) and more likely to attend a cost-free clinic with an aOR 2.72, 95% CI (1.28–5.77). Living far from any town increased health post attendance, with an aOR 1.05 (1.00–1.10) per 1 km further away from the nearest town.

**Table 3:** Therapeutic itinerary in case of fever persistence according to selected determinants on the Thai-Myanmar border

Variable (*n* = distribution in the study population)	**Outcomes: type of healthcare facility attended**														
	**Health post**			**Private clinic**			**Primary care unit**			**Cost-free clinic**			**Hospital**		
	**n (%)**	**aOR**^**a**^** (95%CI)**	***p***	**n (%)**	**aOR**^**a**^** (95%CI)**	***p***	**n (%)**	**aOR**^**a**^** (95%CI)**	***p***	**n (%)**	**aOR**^**a**^** (95%CI)**	***p***	**n (%)**	**aOR**^**a**^** (95%CI)**	***p***
**Country**															
**- Thailand (*n* = 97)**	20 (20.6)	1	< 0.001	51 (52.6)	1	0.008	15 (15.5)	1	0.858	47 (48.5)	1	0.416	26 (26.8)	1	0.071
**- Myanmar (*n* = 103)**	53 (51.5)	2.05 (0.92–4.55)		35 (34.0)	0.69 (0.33–1.42)		15 (14.6)	0.96 (0.35–2.62)		44 (42.7)	1.76 (0.81–3.84)		40 (38.8)	1.41 (0.61–3.21)	
**Cost-free clinic presence in the nearest town**^**b**^															
**- No (*n* = 76)**	42 (55.3)	1	< 0.001	26 (34.2)	1	0.127	14 (18.4)	1	0.337	23 (30.3)	1	*p* < 0.001	38 (50.0)	1	*p* < 0.001
**- Yes (*n* = 119)**	28 (23.5)	0.40 (0.19–0.86)		57 (47.9)	1.53 (0.74–3.17)		16 (13.5)	0.68 (0.25–1.84)		68 (57.1)	2.72 (1.28–5.77)		26 (21.9)	0.31 (0.14–0.67)	
**Distance to nearest town (per + 1 km)**		1.05 (1.00–1.10)	< 0.001		0.97 (0.92–1.01)	0.043		0.94 (0.87–1.02)	0.195		0.96 (0.91–1.00)	0.005		0.99 (0.95–1.04)	0.001
*Legal status*^*c*^															
- *None (n* = *41)*	14 (34.2)	1	0.559	12 (29.3)	1	0.090	5 (12.2)	1	0.572	25 (61.0)	1	0.017	8 (19.5)	1	0.004
- *Unstable (n* = *115)*	45 (39.1)	1.37 (0.57–3.28)		51 (44.4)	1.84 (0.82–4.15)		16 (13.9)	1.03 (0.34–3.19)		53 (46.1)	0.46 (0.20–1.05)		36 (31.3)	1.70 (0.66–4.35)	
- *Stable (n* = *46)*	14 (30.4)	0.94 (0.33–2.64)		24 (52.2)	2.56 (1.00–6.54)		9 (19.6)	1.41 (0.40–4.92)		14 (30.4)	0.27 (0.10–0.71)		24 (52.2)	5.15 (1.80 14.71)	
*Monthly income (in USD)*															
- *Under 90 (n* = *109)*	52 (47.7)	1	0.001	45 (41.3)	1	0.820	16 (14.7)	1	0.950	44 (40.4)	1	0.107	40 (36.7)	1	0.613
- *Between 90–180 (n* = *70)*	17 (24.3)	0.56 (0.26–1.20)		31 (44.3)	0.84 (0.42–1.68)		11 (15.7)	1.03 (0.40–2.69)		39 (55.7)	1.53 (0.75–3.10)		21 (30.0)	0.99 (0.45–2.16)	
- *Over 180 (n* = *23)*	4 (17.4)	0.24 (0.06–0.96)		11 (47.8)	0.76 (0.28–2.08)		3 (13.0)	1.21 (0.28–5.19)		9 (39.1)	0.94 (0.32–2.75)		7 (30.4)	1.10 (0.36–3.33)	

In the first column we presented the distribution of categories for the general population, for each outcome, we present the proportion accessing healthcare at the given health structure

^a^aOR were adjusted by the country of residence, presence of cost-free clinic nearest to participant’s household, legal status and monthly income

^b^Presence of cost-free clinic in the nearest town to the participant’s household included Shoklo Malaria Research Unit (SMRU) clinics and Mae Tao Clinic (MTC)

^c^Legal status was classified as “unstable” for participants owning documents preventing them from healthcare entitlement and freedom of movement. These documents included Myanmar identification card, or a community card, or a hospital card or commuting card. Legal status was classified as “stable” for participants owning documents allowing them to healthcare entitlement and freedom of movement. These documents included work permit, certificate of identity (CI) card, ten-year resident card, Thai identification card, and birth certificate

Participants with a stable legal status were less likely to attend cost-free clinics compared to those without any legal status (aOR 0.27, 95% CI [0.10–0.71]), and attended private clinics and hospitals more often (aOR 2.56, 95% CI [1.00–6.54] and 5.15, 95% CI [1.80–14.71], respectively). Participants earning > USD180 per month were less likely to attend health posts compared to those earning < USD 90 (aOR 0.24, 95% CI [0.06–0.96]).

Phase II participants were also questioned about their main criteria for choosing a given type of health service in case of fever persistence. Consistently with the determinants identified, access to care was declared as the most important criterion for choosing a health service, over direct cost, quality of care, force of habit, word of mouth or management quality (Additional file 3, Table S4).

## Discussion

Our study brought in-depth understanding of fever conception and its believed causes, and identified determinants associated with health-seeking behaviour amongst migrants from the Thai-Myanmar border where the burden of fever is high [5–12]. The only comparable study in the region was carried out in the early 2000s, whereas the economic context has dramatically changed since then, with a USD15 billion China-Thailand-Myanmar Special Economic Zone, and over USD25 billion-a-year gambling industry constructed on the Myanmar side of the border since 2019 [47].

The main barriers on migrant’s journey to care were distance to care and legal status. These two determinants may play a combined role in exposing migrants to a high risk of precarity: distance worsens safety through impractical and flooded tracks and leads to high travel-related costs, while absence of legal status raises security concerns when travelling in areas surrounded by various military and paramilitary groups [48, 49]. In addition, having no legal status is a barrier to secure stable employment, and undocumented migrants are not protected by law nor able to exercise their rights while living and working in Thailand [33]. Health services were apparently diverse on the Thai-Myanmar border but their distribution heterogeneous. In our study, community-level care (i.e., health posts) were only chosen among migrants who were far from all other options. Hospitals were more likely to be attended in areas without cost-free clinics. This confirms distance as a critical factor for choosing the journey to care, dominating other criteria; this finding was consistent elsewhere in rural Asia and Africa [50, 51].

In addition to distance to care and legal status, income was identified as a significant determinant, but only influencing a single type of health service, namely health posts, which are mainly present on the Myanmar side. This lesser role of income level contrasts with the literature [52, 53], and may be related to the particular environment of the Thai-Myanmar border, where cost-free clinics provide free-of-charge care regardless of legal status [54]. In our context, the risk of job loss may overcome income level, as not owning a stable legal status represents a non-negotiable barrier to employment and social protection. On the contrary, income level may be compensated by alternative levers, particularly in rural areas where land, social participation or material possession can provide an economic support [55].

In our study, education-level was not identified as a significant determinant influencing health-seeking behaviour: on the Thai-Myanmar border, governmental and non-governmental organisations (which include Thai public health office, research institute’s running and humanitarian based clinics, ethnic-based health organisations) have been serving the local community for decades, recruiting staff from the same ethnicities and community, therefore disseminating public health messages on illness prevention and detection [56]. Interestingly, a literature review encompassing evidence from both high- and low-to-middle income countries has shown that individual education alone may not be sufficient to change behaviour, and should be accompanied by the concept of “social capital”, as the presence of supportive social groups cooperating to maximise individual’s determinants such as education or even health [57]. It is firmly established in the behaviour change literature that knowledge deficits alone are only a minor driver of behaviour, contributing less than 20% to decision-making process [58, 59]. Nevertheless, a 2019 mixed-methods study carried out collective educational activities, with repeated supervision in three northern-Thai villages on the risk of antibiotic use on antimicrobial resistance (AMR) [60]. Villagers living in a precarious environment were unlikely to change their behaviour even after attending educational sessions, indicating the importance of other factors than education per se.

Our study also highlighted the diversity of language terms referring to fever and its believed causes: such complexity should be investigated prior to any intervention aiming at illness prevention and control. Several surveys highlighted large misunderstandings regarding the terminology related to AMR, both in high- and low-to-middle income countries [61, 62]. In rural northern Thailand, a public health campaign aiming to limit antibiotic use was followed by a drastic reduction of general medicine availability in informal shops, such as paracetamol [60]. This pressurised villagers to seek health in hospital, 9–10 kms away from their village, even for mild symptoms such as headache. Ignoring the language terminology and population beliefs may, therefore, worsen symptom identification and response, while being irrelevant to alleviate the burden of both febrile illness and AMR in Southeast Asia [5–8, 63].

We acknowledge several limitations in our study: the Thai-Myanmar border offers a particular context with substantial health, social and economic inequalities, which limits generalisation of our findings. Our objective, however, was to encourage future behavioural research to incorporate quantitative components for identifying key determinants of health. As a limitation, we could not implement FGD with community members but only with health workers and community representatives, due to logistics reasons. Only benefiting from community member’s perspective in IDIs may have limited our understanding of the diversity of terminology for fever, the corresponding believed causes, and the actions taken towards it. Furthermore, the design of the quantitative part of the study did not include an explicit spatial sampling scheme to ensure representativity across locations. We adjusted for spatial determinants in our analysis regarding the type of health services, but this adjustment might have not been sufficient to remove all confounding effects related to the complexity of the local context. In addition, the number of “nearest towns” where health services could be accessed was limited (six towns, two in Myanmar and four in Thailand), corresponding to four Thai public hospitals and three cost-free clinics. Our analysis did not explore the differences between towns, hospitals or cost-free clinics. For example, public hospitals were regrouped in a unique health service category but are not identical, with local management decisions including hiring Karen-speaking staff or conducting outreach programs. This suggests a more complex relationship between migrants’ determinants and hospital attendance.

Finally, a longitudinal approach with repeated interviews and questionnaires may have strengthened data reliability, incorporating seasonal variations and thus potential changes in health seeking behaviours. Our study took place during the dry season with a concomitant chikungunya outbreak, which probably impacted transport conditions and participant’s risk perception towards their health, therefore influencing their journey to care.

## Conclusion

Combining a qualitative and quantitative approach, our study provides evidence on the determinants influencing health-seeking behaviour in case of fever on the Thai-Myanmar border. We showed the combined importance of multiple factors, ranging from individual representations to availability of health services and geographical, legal and economic barriers. Our findings may guide public health interventions in this region where the burden of fever is substantial: in rural and hard-to-reach areas, health posts are often the only existing structure. Their limited medical skills and treatment options other than testing and treating for malaria may lead to population disinterest, and subsequent emergence of private and often unregulated healthcare. As for migrants living nearer to towns, the apparent larger choice of health services is in fact constrained by multiple non-geographical barriers. Absence of legal status is a major barrier for accessing regulated public healthcare, and initiatives such as migrant health insurance schemes should be encouraged. The importance of cost-free clinics in migrant’s journey to care illustrates how the public health system lacks inclusiveness, especially for those contributing to the region economic development.

## Supplementary Information

**Additional file 1.** BEFIT study topic guide and questionnaire. This additional file contains topic guide used for in-depth interview and focus group discussion (Phase I) and the questionnaire survey used for data collection Phase II of this study, and the questionnaires were used to create an OpenDataKit version (online) to use on tablets.

**Additional file 2: Figure S1.** Geographic distribution of attendance to healthcare services, by nearest town to participants’ house. **Figure S2.** CIT model evaluating demographic and socio-economic determinants for seeking healthcare in case of fever persistence. Hospital, geographic variables. **Figure S3.** CIT model evaluating demographic and socio-economic determinants for seeking healthcare in case of fever persistence. Hospital, socio-demographic and economic variables. **Figure S4.** CIT model evaluating demographic and socio-economic determinants for seeking healthcare in case of fever persistence. NGO free clinic, geographic variables. **Figure S5.** CIT model evaluating demographic and socio-economic determinants for seeking healthcare in case of fever persistence. NGO free clinic, socio-demographic and economic variables. **Figure S6.** CIT model evaluating demographic and socio-economic determinants for seeking healthcare in case of fever persistence. Health post, geographic variables. **Figure S7.** CIT model evaluating demographic and socio-economic determinants for seeking healthcare in case of fever persistence. Health post, socio-demographic and economic variables. **Figure S8.** CIT model evaluating demographic and socio-economic determinants for seeking healthcare in case of fever persistence. Private clinic, geographic variables. **Figure S9.** Diversity of phrases or set of terms to describe fever.

**Additional file 3: Table S1.** Fever terminology among Karen and non-Karen participants on the Thai-Myanmar border. **Table S2.** Health-seeking behaviour at fever onset according to all determinants from 200 Phase II participants Of 202 in total, 2 participants reporting an “Other” behaviour are not shown here. Each participant could provide only one response. **Table S3.** Health-seeking behaviour in case of fever persistence according to all determinants from Phase II participants (*n* = 202). Participants could report attending multiple facilities. **Table S4.** Declared criteria of healthcare service preference in case of fever persistence among Phase II participants.

**Additional file 4.** Supplementary study information. The PDF document contains additional information, and insights of the study, which include What is already known? What are the new findings? What do the new findings imply? Strength and Limitations of the study.

## Data Availability

All relevant qualitative data are presented within the paper. Due to ethical reasons, and to protect confidentiality and privacy research participants, the complete interview data cannot be made available to the public. A list of condensed meaning units or codes can be made available upon reasonable request to the Data Access Committee of Mahidol Oxford Tropical Medicine Research Unit (MORU). For more information, please see MORU Tropical Network Policy on Sharing Data and Other Outputs. Quantitative statistical datasets supporting this article and the conclusions of this article is included within the article and its Additional files 2 and 3. Participatory Visual Method video output generated during the study is available in the figshare repository, https://doi.org/10.6084/m9.figshare.11481141.v1.

## Abbreviations

AMR: Antimicrobial resistance
aOR: Adjusted Odd Ratio
ACT: Artemisinin-based combined therapy
CI: Confidence interval
CIT: Conditional Inference Tree
MTC: Mae Tao Clinic
FGD: Focus Group Discussion
HW: Health worker
IDI: In-depth interview
IRD: Institut de Recherche & Développement
INSERM: Institut National de la Santé et de la Recherche Médicale
IQR: Interquartile range
MKT: Mo Ker Thai clinic
ODK: OpenDataKit
PVM: Participatory Visual Method
PCU: Primary Care Unit
PCU staff: Primacy Care Unit staff
SESSTIM: Sciences Économiques et Sociales de la Santé & Traitement de l’Information Médicale
SMRU: Shoklo Malaria Research Unit
SD: Standard Deviation
T-CAB: Tak Province Community Ethics Advisory Board
USD: United States Dollar
VHV: Village Health Volunteer
