# Supplementary material for: Fever and health-seeking behaviour among migrants living along the Thai-Myanmar border: a mixed-methods study
Source: BMC Infect Dis. 2023 Jul 31;23:501. doi: 10.1186/s12879-023-08482-8 (PMC10388507; doi:10.1186/s12879-023-08482-8)
Supplement: Supplementary file 1 — Additional file 1. BEFIT study topic guide and questionnaire. This additional file contains topic guide used for in-depth interview and focus group discussion (Phase I) and the questionnaire survey used for data collection Phase II of this study, and the questionnaires were used to create an OpenDataKit version (online) to use on tablets. [file 12879_2023_8482_MOESM1_ESM.pdf]

Topic Guide for individual in-depth interviews (IDIs) and focus group discussions (FGDs)

Study: Health seeking behaviours during acute febrile illnesses in Tak province (Phase 1 qualitative research)

Key Objective To understand health-seeking behaviours among community members living in rural and remote areas in Myanmar and along the Thai-Myanmar border, given the context of acute febrile illness.

Objectives

- To engage with community healthcare workers (CHW) and primary care staffs in order to understand:
  - Their role within the community
  - The constraints and context they operate under
  - Their management of fever including that related to respiratory and abdominal complaints
- To engage with community members and their representatives living in rural and remote along the Thai-Myanmar border, both Thailand and Myanmar sides in order to understand:
  - Their conception of fever, including that related to respiratory and abdominal complaints
  - Their conception of the potential causes of fever, including that related to respiratory and abdominal complaints
  - Their health-seeking behaviours and their determinants during the occurrence of fever, including that related to respiratory and abdominal complaints

Questions

Conception and understanding of fever

- In your opinion, what is fever, febrile illness including respiratory and digestive problems or symptoms? Can you please share what is fever for you? Can you describe what is fever or when you have fever?
- What (physical) symptoms do you have when you feel feverish?
- How do you think you get fever/symptoms?

Health seeking behaviors

- Can you share with us what do you do then when you have these symptoms? (Probe: for example, do you seek help from anyone? Who do you consult? How long do you wait before going to seek health care (going to pharmacies, traditional healers, doctors?)
- Why do you choose these choices of health care? (Cheap, convenient, affordable?)

- Is there any public hospital around where you live/work? Why do you/ don't you go to this public hospital? What are barriers and enablers to access public health care?

Specific questions to:
<u>Community Healthcare Workers</u>
To engage with community healthcare workers (CHW) and primary care staffs in order to understand

- What are your roles towards the community-members and the local health system?
- In your opinion, what is fever, febrile illness including respiratory and digestive problems or symptoms? Can you please share what is fever for you?
- How frequently do you manage febrile patients?
- In your current job, what are the challenges you encounter on daily basis? In particular, what challenges to you face in your management of febrile patients? In what way?

<u>Key informants</u>

To get their view and background understanding from key informants from communities such as village chief, elders etc.

- What are your roles towards the community-members?
- In your opinion, what is fever, febrile illness including respiratory and digestive problems or symptoms? Can you please share what is fever for you?
- What do people in your community normally do when they have fever? Where do they go to seek help or treatment?
- Have any community members come to your to seek your help when they fall sick, especially when they have fever? What kind of help/support have they asked for? What kind of support have you provided?
- Is there any public hospital around your community? What types of healthcare facilities are available in your area? Why do/don't people in your community go to this public hospital? What are barriers and enablers to access public health care?

Questionnaire with question guidance & conditioning

Study: Health seeking behaviours during acute febrile illnesses in Tak province (Phase 2 quantitative research)

Participants should consent prior starting this questionnaire. Make clear that all information collected will be anonymously recorded and stored without being divulgated.

In case the answer "Others, specify: ______" is too long for the electronic questionnaire, the interviewer should report the long answer on a separate sheet of paper with the corresponding participant identifier, and report it to Napat on the same day of the collection for choosing the best way to report the answer.

All options of each question should be cited to the participant.

1. Age? ____ (years)

2. Sex?
    a. Female
    b. Male

3. Marital status? (unique choice)
    a. Single
    b. Married
    c. Separated/Divorced
    d. Widowed/widower

4. Religion/belief? (multiple choices allowed)
    a. Christian
    b. Buddhist
    c. Animism
    d. Muslim
    e. Others, specify: _________

5. Ethnicity? (unique choice)
    a. Karen
    b. Burmese
    c. Thai-Karen
    d. Others, specify: _________

6. Level of education completed (unique choice)
    a. Informal (in monastery, in camp, in family, but without official teacher)
    b. Primary school (grade 1 to 4)
    c. Secondary school (grade 5 to 10)
    d. Post 10 (university)
    e. No education
    f. Others, specify: _________

7. Where do you live now? ______ (village)

8. How long have you lived there? _______year_______ month

9. How many people do you live with? _________ person(s)

10. Who are you currently living with?
    a. Alone
    b. With other people

    10a. Who are you currently living with? (multiple choices allowed)
    c. Parents, Sibling(s) or relatives
    d. Friend(s)
    e. Child/children (under 15)
    f. Spouse
    g. Others, specify: _________

11. What is your main source of income? (unique choice)
    a. Daily labor
    b. Farmer/farming (own or renting land for farming)
    c. Domestic work (maid)
    d. No income
    e. Others, specify: _________

12. Do you have another job?
    Here we aim to know whether there is any additional income, especially non-monetary income such as production of honey, milk, meat, bread, vegetables, fishing, craft that the participant can trade
    a. No secondary income
    b. Yes, please specify: _________

13. What is your family monthly approximate income? (unique choice)
    Interviewer should estimate both the financial and the non-financial income, on a monthly basis. This income may vary by month, and the interviewer should be able to estimate the average income
    a. Below 3,000 THB
    b. Between 3,000 to 6,000 THB
    c. Between 6,000 to 10,000 THB
    d. Between 10,0000 to 15,000 THB
    e. Above 15,000 THB

14. Have you ever heard about vaccination? (unique choice)
    a. Yes
    b. No

15. What do you understand about vaccination? (multiple choices allowed)

In case the participant heard of vaccination but does choose answer "Other, specify", the interviewer should aim to understand whether the participant has a positive or negative opinion about vaccination.

a. For good health
b. To protect infectious diseases
c. Makes people sick
d. I don't understand about it
e. Others, specify: _________________

16. Do you have any of following documents? (multiple choices allowed)
    a. Myanmar Passport/ID
    b. Thai national ID
    c. Birth certificate (different than Delivery certificate)
    d. Health insurance, if yes, specify: _______
    e. Hospital card
    f. 10-year card
    g. 250-baht card for commuting in the area
    h. CI
    i. Work permit
    j. No documents
    k. Others, specify: _________

<u>Conception of fever and causes of fever</u>

Please explain to the participant the objective of the following questions: "We would like to know what is fever for you, the words you use to define it, and the actions you take when it occurs."

17. What are the symptoms when you have fever? (multiple choices allowed)
    a. Hot and cold body
    b. Body ache/joint pain/muscle pain
    c. Feeling discomfort
    d. Headache
    e. Skin rashes
    f. Cough
    g. Runny nose
    h. Sore throat
    i. Diarrhea/stomach ache
    j. Urinary symptoms/burn
    k. Others, specify: _________

18. From all the answers above, what do you call it in your native language? (multiple choices allowed)
    In this question, we aim to know what term the participant uses to define "fever". There may be multiple terms so the question should be formulated to encourage the participant to provide all the potential words commonly used to define "fever"
    a. It's fever/hot body
    b. It's (*ta nya ghoe)* တၢ်ညၣ်ဂိၢ်
    c. It's dengue "htohgway" တုတ်ကွေ

d. It’s malaria
e. Others, specify: _________

19. How many times last year have you experienced fever? (unique choice)
    a. Never
    b. Once
    c. Twice
    d. More than twice (specify): _____(number)
    e. Can’t recall
        i. Too frequent
        ii. Quite rare

20. How do you get fever? (multiple choices allowed)
    Here we aim to know what are the main causes of fever. How can the participant catch fever?
    a. Bug/virus got into the body
    b. Walking in the rain/shower late at night
    c. Bitten by mosquitos
    d. Chikungunya, Dengue, Common cold, Influenza
    e. Boil/abscess
    f. Stepping on nail/ infected wound
    g. Respiratory infection, ear infection, tonsillitis, pneumonia, sore throat
    h. Urine infection
    i. Poisonous food, stomach ache, diarrhea
    j. Exhaustion/intensive work
    k. Spirits
    l. Others, specify: _________

Fever and therapeutic itinerary

21. What do you do first when do you have fever? (unique choice)
    Here the participant may want to answer multiple answers. We want to know what is the most common behavior, the most frequent action the participant takes when fever just occurs in the first days. If the participant answers “a” and “b” or “a” and “c”, or if the participant admits going rapidly to the grocery shops or taking medicine from home if symptoms persist, then the interviewer should not consider “a” as the final answer.
    a. Wait and see
    b. Taking medicine from grocery shops
    c. Taking medicine available at home/neighbor/friends
    d. Taking traditional herbal medicine/seeing traditional healers
    e. Seeing *mor kapao (unqualified health worker)*
    f. Going to see doctors at clinic, PCU, hospital
    g. Seeking help from a trained health worker or health post (village health volunteer or foreign health volunteer)
    h. Others, specify: _________

22. Why do you choose that first? (multiple choices allowed)
    a. Hope to get better from resting at home
    b. It's close by
    c. It's cheap
    d. It's free
    e. Because the quality of the service
    f. Others, specify: _________

23. What main medicine do you take by yourself when you have fever? (multiple choices allowed)
    In this question, we want the main drugs participant is used to taking when fever. We don't want all fo them, just the most frequently taken in case of fever.
    a. Paracetamol (Decolgen, Tiffy, Tylinol, Sara)
    b. *Ya chood*
    c. Antibiotic (Amoxy, Augmentin or Amoxiclav, etc)
    d. Anti-inflammatory (Bufen, Ibuprofen, Gofen)
    e. Burmese herbal medicine
    f. Anti-malarial
    g. Color of drug (any color) ________
    h. Do not know
    i. Others, specify: ________

24. Where do you get them? (multiple choices allowed)
    a. I have it at home
    b. Grocery shop
    c. Pharmacy
    d. Neighbors/friends
    e. *Mor kapao* (unqualified health worker)
    f. Other, specify_________________

25. If fever persists, what do you do? (multiple choices allowed)
    Each option (health worker/health post; private clinic; public clinic; hospital…) should be cited to the participant.
    a. Going to health worker/Health post
        i. Yes
            1. Reasons
                - Because it is convenient to travel to.
                - Because it is free/affordable.
                - Because of the quality/types of service that the facility provides.
                - Because I used to go there.
                - Word of mouth
                - Because the staff treat me well.
                - Others, specify: _________

        ii. No
            1. Reasons

- It's too far
- It's too expensive
- They do not offer good service
- I never went there before
- It has a bad reputation
- The staff was not nice to me
- Others, specify: _________

b. Going to a private clinic (pay) (if yes, where)

1. Myanmar
2. Thailand

ii. Yes

iii. Reasons

1. Because it is convenient to travel to.
2. Because it is free/affordable.
3. Because of the quality/types of service that the facility provides.
4. Because I used to go there.
5. Word of mouth
6. Because the staff treat me well.
7. Others, specify: _________

iv. No

v. Reasons

1. It's too far
2. It's too expensive
3. They do not offer good service
4. I never went there before
5. It has a bad reputation
6. The staff was not nice to me
7. Others, specify: _________

c. Going to PCU (*ana-mai*)/RHC (rural health centre)

i. Yes

ii. Reasons

1. Because it is convenient to travel to.
2. Because it is free/affordable.
3. Because of the quality/types of service that the facility provides.
4. Because I used to go there.
5. Word of mouth
6. Because the staff treat me well.
7. Others, specify: _________

iii. No

iv. Reasons

1. It's too far

2. It's too expensive
3. They do not offer good service
4. I never went there before
5. It has a bad reputation
6. The staff was not nice to me
7. Others, specify: _________

d. Going to public hospital, if yes where?
   1. Myanmar
   2. Thailand

ii. Yes

iii. Reasons
   1. Because it is convenient to travel to.
   2. Because it is free/affordable.
   3. Because of the quality/types of service that the facility provides.
   4. Because I used to go there.
   5. Word of mouth
   6. Because the staff treat me well.
   7. Others, specify: _________

iv. No

v. Reasons
   1. It's too far
   2. It's too expensive
   3. They do not offer good service
   4. I never went there before
   5. It has a bad reputation
   6. The staff was not nice to me
   7. Others, specify: _________

e. Going to SMRU/Mae Tao Clinic

i. Yes

ii. Reasons
   1. Because it is convenient to travel to.
   2. Because it is free/affordable.
   3. Because of the quality/types of service that the facility provides.
   4. Because I used to go there.
   5. Word of mouth
   6. Because the staff treat me well
   7. Others, specify: _________

iii. No

iv. Reasons
   1. It's too far
   2. It's too expensive
   3. They do not offer good service

4. I never went there before
5. It has a bad reputation
6. The staff was not nice to me
7. Others, specify: _________

26. In case of fever persistence, after how many days do you wait before going to …? (unique choice)
In case of fever persistence, the participant named the facility(ies) he/she prefers to go to. We want to know how long he/she waits before going.
    a. 1-2 days
    b. 2-3 days
    c. 3 and more days
    d. Others, specify: _________

27. Why do you wait? (multiple choices allowed)
    a. I want to wait and see at home (resting).
    b. I believe that I can self-treat by taking some medicine available at home.
    c. It's too far to travel to clinics/PCU/hospital.
    d. It's expensive/unaffordable for me.
    e. I don't want to miss work.
    f. I don't want to see doctors.
    g. There are no health facilities near my place.
    h. Others, specify: _________

28. Do you prefer shots or oral medicine? (unique choice)
    a. Injections
    b. Oral medicine
    c. Unsure/don't know

29. Why do you prefer the medicine chosen above? (multiple choices allowed)
    a. It's fast for recovery (high efficacy)
    b. It works before
    c. I am used to it
    d. It's more affordable
    e. Others, specify: _________
