# Supplementary material for: Fever and health-seeking behaviour among migrants living along the Thai-Myanmar border: a mixed-methods study
Source: BMC Infect Dis. 2023 Jul 31;23:501. doi: 10.1186/s12879-023-08482-8 (PMC10388507; doi:10.1186/s12879-023-08482-8)
Supplement: Supplementary file 2 — Additional file 2: Figure S1. Geographic distribution of attendance to healthcare services, by nearest town to participants’ house. Figure S2. CIT model evaluating demographic and socio-economic determinants for seeking healthcare in case of fever persistence. Hospital, geographic variables. Figure S3. CIT model evaluating demographic and socio-economic determinants for seeking healthcare in case of fever persistence. Hospital, socio-demographic and economic variables. Figure S4. CIT model evaluating demographic and socio-economic determinants for seeking healthcare in case of fever persistence. NGO free clinic, geographic variables. Figure S5. CIT model evaluating demographic and socio-economic determinants for seeking healthcare in case of fever persistence. NGO free clinic, socio-demographic and economic variables. Figure S6. CIT model evaluating demographic and socio-economic determinants for seeking healthcare in case of fever persistence. Health post, geographic variables. Figure S7. CIT model evaluating demographic and socio-economic determinants for seeking healthcare in case of fever persistence. Health post, socio-demographic and economic variables. Figure S8. CIT model evaluating demographic and socio-economic determinants for seeking healthcare in case of fever persistence. Private clinic, geographic variables. Figure S9. Diversity of phrases or set of terms to describe fever. [file 12879_2023_8482_MOESM2_ESM.pdf]

Additional file 2 – Supplementary figures

Study title: Fever and health-seeking behaviour among migrants living along the Thai-Myanmar border: a mixed-methods study

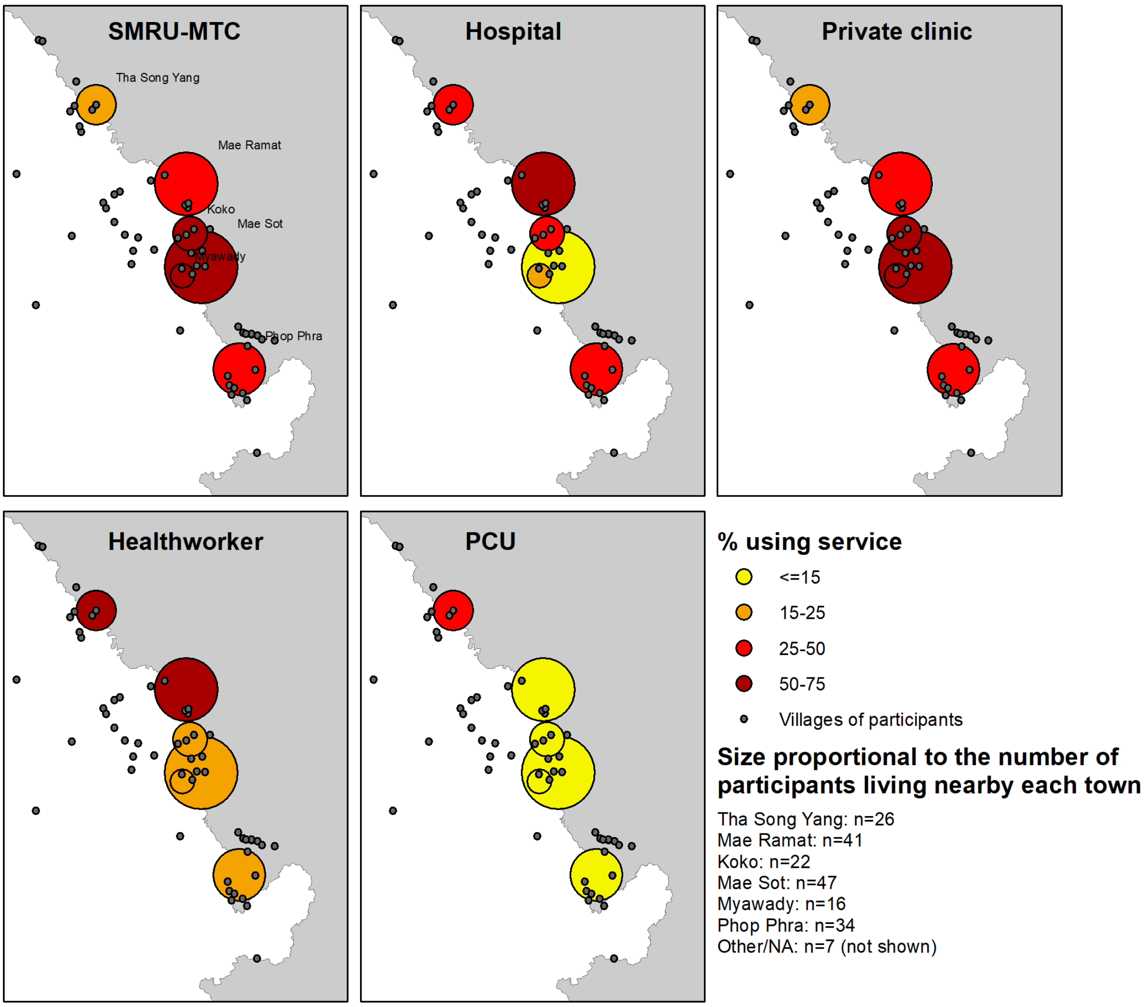

Figure S1. Geographic distribution of attendance to healthcare services, by nearest town to participants' house.

Additional file 2 – Supplementary figures

Study title: Fever and health-seeking behaviour among migrants living along the Thai-Myanmar border: a mixed-methods study

The **Figure S2 – Figure S8** are of CIT model evaluating demographic and socio-economic determinants for seeking healthcare in case of fever persistence, by type of healthcare service and variables.

Geographic variables: distance to nearest town, presence of an NGO clinic (cost-free clinics, which are SMRU clinics, or Mae Tao Clinic) in the nearest town or hospital only, and country of residence.

Socio-demographic and economic variables: sex, ethnicity, income, legal status, main activity, religion, age, education and number of household members.

N.B.: no demographic nor socio- economic variables were identified for private clinics and primary care units (PCUs).

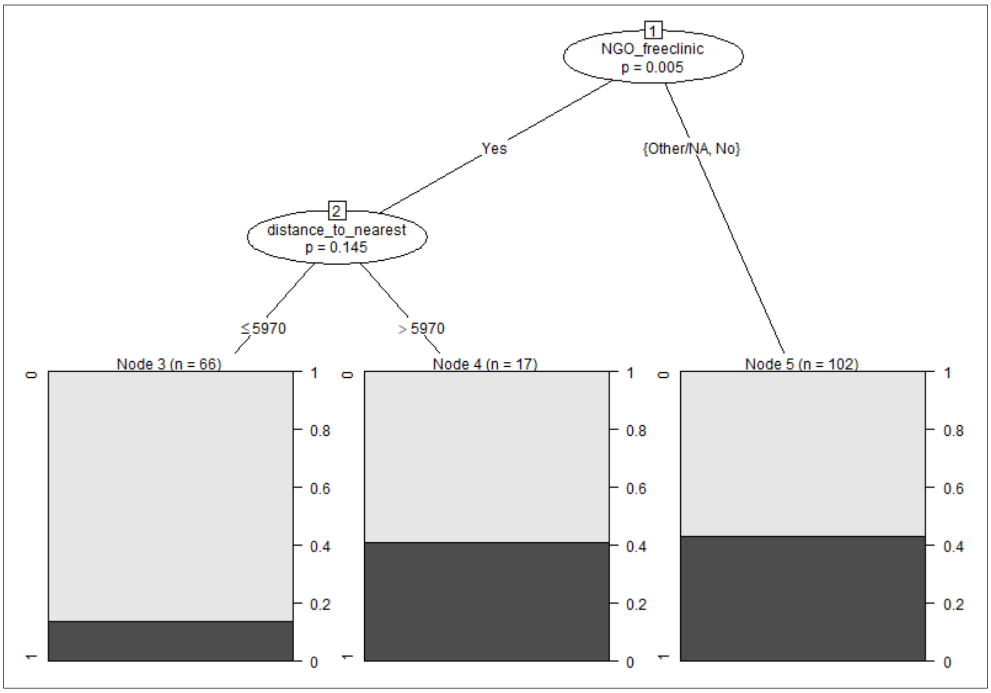

Figure S2. CIT model evaluating demographic and socio-economic determinants for seeking healthcare in case of fever persistence. Hospital, geographic variables.

Additional file 2 – Supplementary figures

Study title: Fever and health-seeking behaviour among migrants living along the Thai-Myanmar border: a mixed-methods study

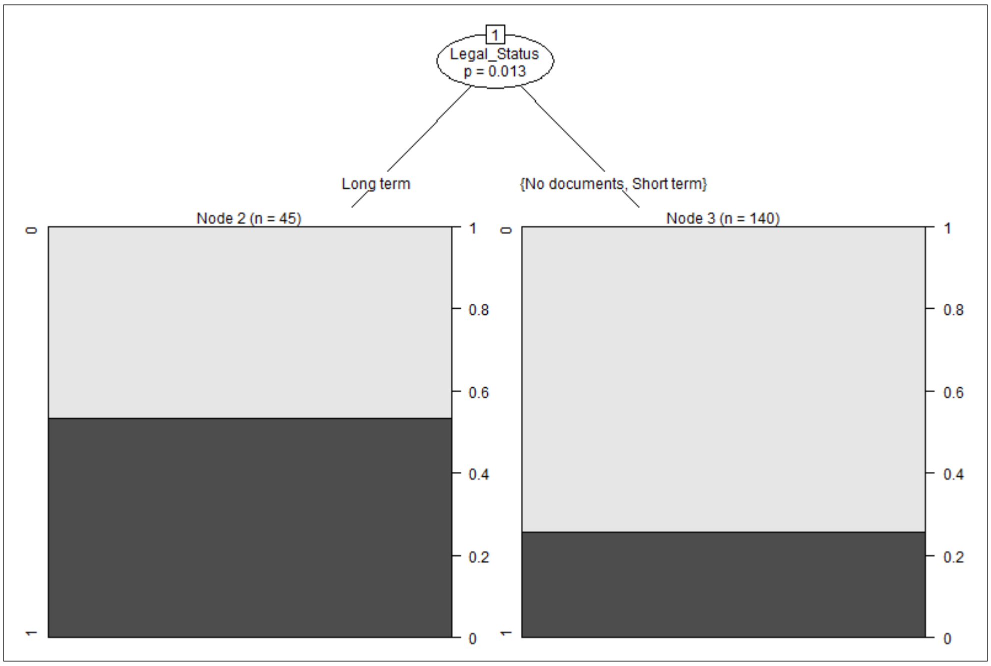

Figure S3. CIT model evaluating demographic and socio-economic determinants for seeking healthcare in case of fever persistence. Hospital, socio-demographic and economic variables.

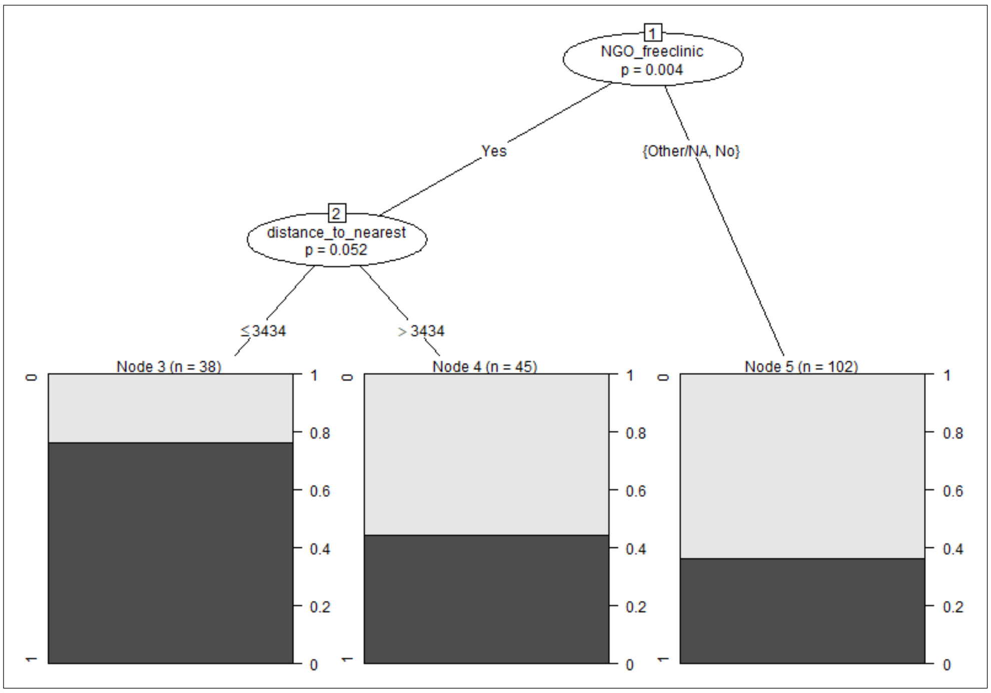

Figure S4. CIT model evaluating demographic and socio-economic determinants for seeking healthcare in case of fever persistence. NGO free clinic, geographic variables.

Study title: Fever and health-seeking behaviour among migrants living along the Thai-Myanmar border: a mixed-methods study

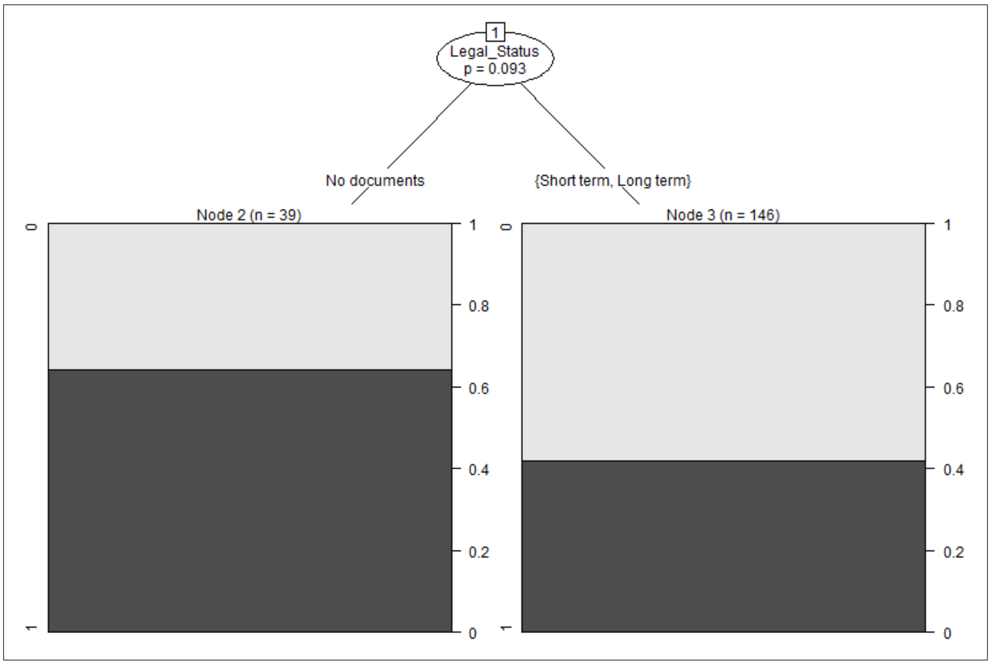

Figure S5. CIT model evaluating demographic and socio-economic determinants for seeking healthcare in case of fever persistence. NGO free clinic, socio-demographic and economic variables.

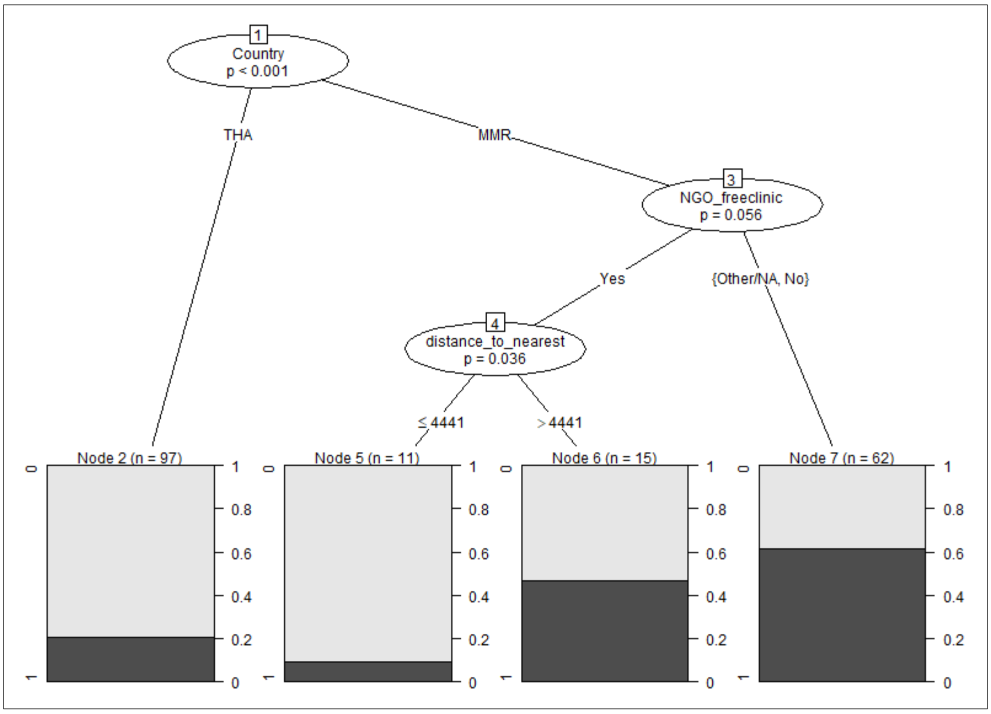

Figure S6. CIT model evaluating demographic and socio-economic determinants for seeking healthcare in case of fever persistence. Health post, geographic variables.

Additional file 2 – Supplementary figures

Study title: Fever and health-seeking behaviour among migrants living along the Thai-Myanmar border: a mixed-methods study

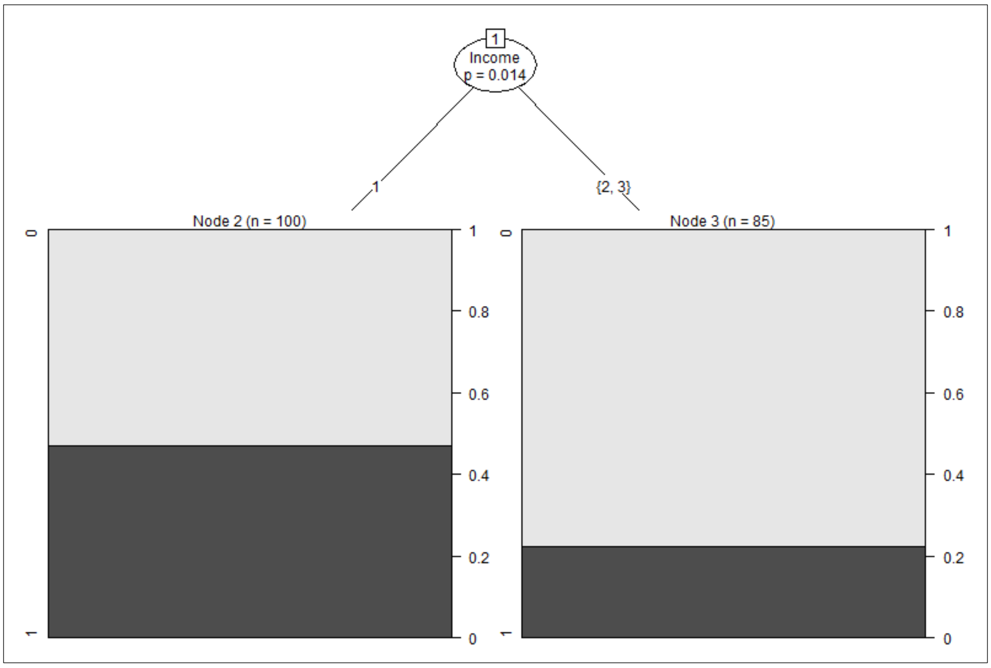

Figure S7. CIT model evaluating demographic and socio-economic determinants for seeking healthcare in case of fever persistence. Health post, socio-demographic and economic variables.

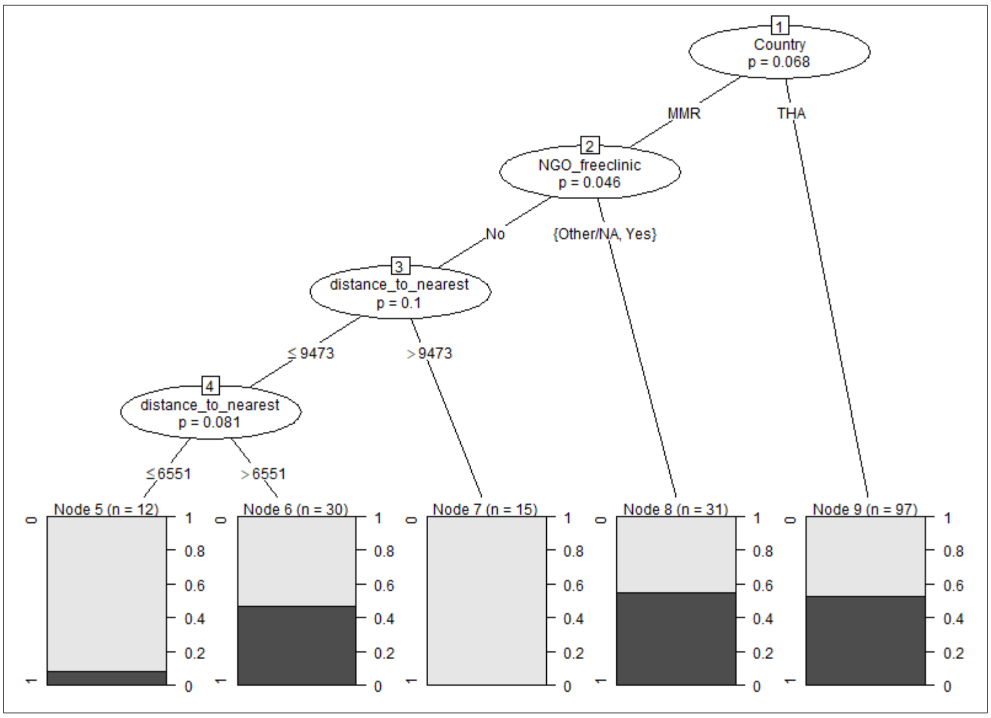

Figure S8. CIT model evaluating demographic and socio-economic determinants for seeking healthcare in case of fever persistence. Private clinic, geographic variables.

Study title: Fever and health-seeking behaviour among migrants living along the Thai-Myanmar border: a mixed-methods study

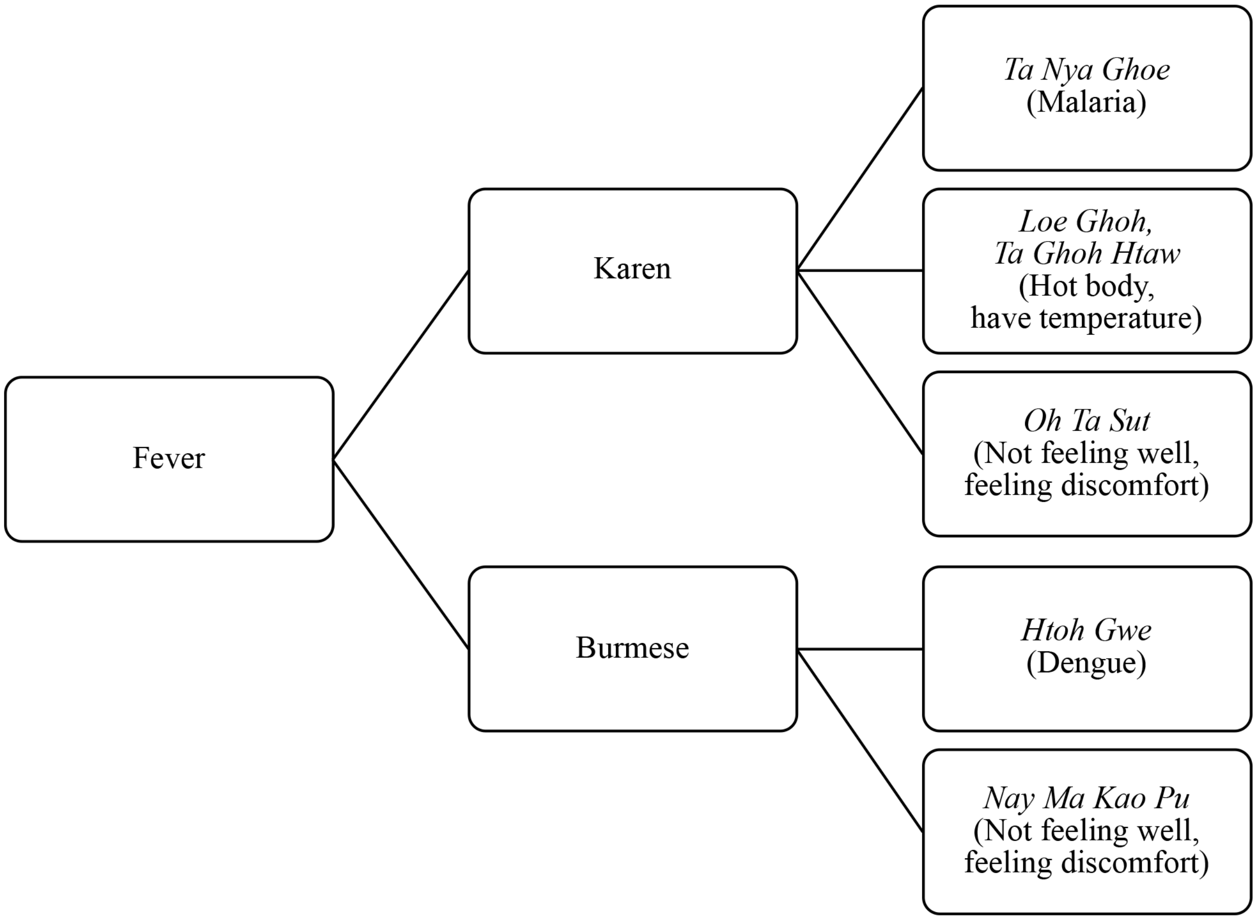

Figure S9. Diversity of phrases or set of terms to describe fever.
