# Supplementary material for: Fever and health-seeking behaviour among migrants living along the Thai-Myanmar border: a mixed-methods study
Source: BMC Infect Dis. 2023 Jul 31;23:501. doi: 10.1186/s12879-023-08482-8 (PMC10388507; doi:10.1186/s12879-023-08482-8)
Supplement: Supplementary file 3 — Additional file 3: Table S1. Fever terminology among Karen and non-Karen participants on the Thai-Myanmar border. Table S2. Health-seeking behaviour at fever onset according to all determinants from 200 Phase II participants Of 202 in total, 2 participants reporting an “Other” behaviour are not shown here. Each participant could provide only one response. Table S3. Health-seeking behaviour in case of fever persistence according to all determinants from Phase II participants (n = 202). Participants could report attending multiple facilities. Table S4. Declared criteria of healthcare service preference in case of fever persistence among Phase II participants. [file 12879_2023_8482_MOESM3_ESM.pdf]

Additional file 3 – Supplementary tables

Study title: Fever and health-seeking behaviour among migrants living along the Thai-Myanmar border: a mixed-methods study

Table S1. Fever terminology among Karen and non-Karen participants on the Thai-Myanmar border.

**Fever terminology**	Non- Karen* (n=68)	Karen (n=134)	p-value
"Fever"	40 (58.8)	53 (39.6)	0.009
Ta Nya Ghoe	0	28 (20.9)	<0.001
Dengue	4 (5.9)	11 (8.2)	0.551
Malaria	2 (2.9)	4 (3.0)	0.986
Oh Ta Sut	1 (1.5)	3 (2.2)	0.711
"Sick"	21 (30.9)	48 (35.8)	0.484
Name depends on the symptoms	8 (11.8)	9 (6.7)	0.222
Other	1 (1.5)	6 (4.5)	0.270
At least two terms referring to fever	9 (13.2)	30 (22.4)	0.119
Three terms referring to fever	0	4 (3.0)	0.150

*Non-Karen ethnicity included Burmese (n=51), Pa-Oh (n=12), Mon (n=2), Shan (n=1), Chin (n=1), and one participant from Rakhine state (ethnicity unknown)

Study title: Fever and health-seeking behaviour among migrants living along the Thai-Myanmar border: a mixed-methods study

Table S2. Health-seeking behaviour at fever onset according to all determinants from 200 Phase II participants Of 202 in total, 2 participants reporting an "Other" behaviour are not shown here. Each participant could provide only one response.

	**Wait & See N=16**	**Self-medication N=124**	**Unregulated healthcare N=29**	**Regulated healthcare N=31**
Demographic & Geographical determinants				
Age (in years)	35 (29-44)	32 (26-43)	35 (30-49)	29 (25-36)
Female (n=127)	10 (7.9)	78 (61.4)	17 (13.4)	22 (17.3)
Married (n=186)	13 (7.0)	114 (61.3)	29 (15.6)	30 (16.1)
Religion				
- Buddhist (n=172)	13 (7.6)	109 (63.4)	24 (14.0)	26 (15.1)
- Christian (n=26)	3 (11.5)	14 (53.8)	5 (19.2)	4 (15.4)
- Other[*] (n=2)	0	1 (50.0)	0	1 (50.0)
Ethnicity				
- Karen (n=132)	14 (10.6)	82 (62.1)	15 (11.4)	21 (15.9)
- Burmese (n=51)	2 (3.9)	31 (60.8)	10 (19.6)	8 (15.7)
- Other[**] (n=17)	0	11 (64.7)	4 (23.5)	2 (11.8)
Education level				
- None/Informal (n=66)	6 (9.1)	42 (63.6)	7 (10.6)	11 (16.7)
- Primary (n=67)	6 (9.0)	37 (55.2)	13 (19.4)	11 (16.4)
- Secondary or above (n=67)	4 (6.0)	45 (67.2)	9 (13.4)	9 (13.4)
Country				
- Thailand (n=96)	6 (6.3)	59 (61.5)	19 (19.8)	12 (12.5)
- Myanmar (n=102)	9 (8.8)	64 (62.8)	10 (9.8)	19 (18.6)
Presence of a NGO clinic in the nearest town [***]				
- No (n=76)	9 (11.8)	48 (63.2)	9 (11.8)	10 (13.2)
- Yes (n=117)	5 (4.3)	72 (61.5)	19 (16.2)	21 (18.0)
Distance to nearest town (in km)	8.4 (7.2-8.7)	6.9 (2.6-9.4)	7.4 (2.3-13.2)	6.6 (3.4-16.6)

Study title: Fever and health-seeking behaviour among migrants living along the Thai-Myanmar border: a mixed-methods study

Socio-economic determinants				
Number of people in the household	4 (4-5)	4 (3-5)	3 (3-5)	5 (3-6)
Legal status****				
- None (n=40)	2 (5.0)	25 (62.5)	4 (10.0)	9 (22.5)
- Unstable (n=114)	9 (7.9)	69 (60.5)	17 (14.9)	19 (16.7)
- Stable (n=46)	5 (10.9)	30 (65.2)	8 (17.4)	3 (6.5)
Principal activity				
- Daily labour (n=74)	6 (8.1)	41 (55.4)	15 (20.3)	12 (16.2)
- Farmer (n=68)	4 (5.9)	46 (67.7)	9 (13.2)	9 (13.2)
- Teacher/Health worker	2 (16.7)	7 (58.3)	1 (6.3)	2 (18.8)
- None/Domestic (n=46)	4 (8.7)	30 (65.2)	4 (8.7)	8 (17.4)
Monthly income (in USD)				
- Under 90 (n=108)	10 (9.3)	68 (63.0)	15 (13.9)	15 (13.9)
- Between 90-180 (n=69)	4 (5.8)	45 (65.2)	7 (10.1)	13 (18.8)
- Over 180 (n=23)	2 (8.7)	11 (47.8)	7 (30.4)	3 (13.0)

N.B. The table shows median (IQR) for three numeric variables (age, distance to the nearest town, number of people in the household) and number (%) for categorical variables (all others).

*Other religion included Islam (n=1) and agnostic (n=1)
**Other ethnicity included Pa-oh (n=12), Chin (n=1), Hmong (n=2), Shan (n=1), and a participant from Rakhine state, Myanmar (n=1)
***Presence of an NGO clinic, in the town nearest to the residence of participant, which include Shoklo Malaria Research Unit (SMRU) clinics and Mae Tao Clinic. *Healthcare services provided at these clinics are free-of-charge.*
****Legal status was classified as "unstable" for participants owning documents preventing them from healthcare entitlement and freedom of movement. These documents included Myanmar identification card, or a community card, or a hospital card or commuting card. Legal status was classified as "stable" for participants owning documents allowing them to healthcare entitlement and freedom of movement. These documents included work permit, certificate of identity (CI) card, ten-year resident card, Thai identification card, birth certificate.

Options listed of health seeking behaviour at fever onset originate from Phase I qualitative analysis

Study title: Fever and health-seeking behaviour among migrants living along the Thai-Myanmar border: a mixed-methods study

Table S3. Health-seeking behaviour in case of fever persistence according to all determinants from Phase II participants (n=202). Participants could report attending multiple facilities.

	**Health post N=73**	**Private clinic N=87**	**Primary care unit N=30**	**NGO clinic N=92**	**Hospital N=68**
Demographic & Geographic determinants					
Age (in years)	30 (25-42)	32 (26-45)	33 (27-41)	30 (25-38)	30 (25-37)
Female (n=129)	41 (31.8)	63 (48.8)	20 (15.5)	60 (46.5)	39 (30.2)
Married (n=188)	64 (34.0)	82 (43.6)	28 (14.9)	89 (47.3)	63 (33.5)
Religion					
- Buddhist (n=174)	60 (34.5)	78 (44.8)	25 (14.4)	80 (46.0)	57 (32.8)
- Christian (n=26)	12 (46.2)	8 (30.8)	5 (19.2)	11 (42.3)	10 (38.5)
- Other* (n=2)	1 (50.0)	1 (50.0)	0	1 (50.0)	1 (50.0)
Ethnicity					
- Karen (n=134)	59 (44.0)	50 (37.3)	22 (16.4)	61 (45.5)	50 (37.3)
- Burmese (n=51)	10 (19.6)	26 (51.0)	6 (11.8)	22 (43.1)	11 (21.6)
- Other** (n=17)	4 (23.5)	11 (64.7)	2 (11.8)	9 (52.9)	7 (41.2)
Education level					
- None/Informal (n=67)	23 (34.3)	25 (37.3)	9 (13.4)	28 (41.8)	19 (28.4)
- Primary (n=68)	18 (26.5)	32 (47.1)	11 (16.2)	35 (51.5)	22 (32.4)
- Secondary or above [illegible]	32 (47.8)	30 (44.8)	10 (14.9)	29 (43.3)	27 (40.3)
Country					
- Thailand (n=97)	20 (20.6)	51 (52.6)	15 (15.5)	47 (48.5)	26 (26.8)
- Myanmar (n=103)	53 (51.5)	35 (34.0)	15 (14.6)	44 (42.7)	40 (38.8)
Presence of a NGO clinic in the nearest town ***					
- No (n=76)	42 (55.3)	26 (34.2)	14 (18.4)	23 (30.3)	38 (50.0)
- Yes (n=119)	28 (23.5)	57 (47.9)	16 (13.5)	68 (57.1)	26 (21.9)
Distance to nearest town (in km)	8.4 (5.7-13.2)	6.8 (2.6-9.4)	6.3 (2.6-8.4)	6.0 (1.5-8.4)	8.4 (6.8-11.3)

Socio-economic determinants					
Number of people in the household	4 (3-5)	4 (3-5)	4 (3-6)	4 (3-5)	4 (3-6)
Legal status****					
- None (n=41)	14 (34.2)	12 (29.3)	5 (12.2)	25 (61.0)	8 (19.5)
- Unstable (n=115)	45 (39.1)	51 (44.4)	16 (13.9)	53 (46.1)	36 (31.3)
- Stable (n=46)	14 (30.4)	24 (52.2)	9 (19.6)	14 (30.4)	24 (52.2)
Principal activity					
- Daily labour (n=75)	19 (25.3)	31 (41.3)	11 (14.7)	30 (40.0)	18 (24.0)
- Farmer (n=68)	27 (39.7)	28 (41.2)	8 (11.8)	30 (44.1)	32 (47.1)
- Teacher/Health worker [illegible]	7 (45.5)	5 (35.7)	2 (16.7)	7 (58.3)	4 (33.3)
- None/Domestic (n=47)	20 (42.6)	24 (51.1)	9 (19.1)	25 (53.2)	14 (29.8)
Monthly income (in USD)					
- Under 90 (n=109)	52 (47.7)	45 (41.3)	16 (14.7)	44 (40.4)	40 (36.7)
- Between 90-180 (n=70)	17 (24.3)	31 (44.3)	11 (15.7)	39 (55.7)	21 (30.0)
- Over 180 (n=23)	4 (17.4)	11 (46.8)	3 (13.0)	9 (39.1)	7 (30.4)

N.B. The table shows median (IQR) for three numeric variables (age, distance to the nearest town, number of people in the household) and number (%) for categorical variables (all others).

*Other religion included Islam (n=1) and agnostic (n=1)
**Other ethnicity included Pa-oh (n=12), Chin (n=1), Hmong (n=2), Shan (n=1), and a participant from Rakhine state, Myanmar (n=1)
***Presence of an NGO clinic, in the town nearest to the residence of participant, which include Shoklo Malaria Research Unit (SMRU) clinics and Mae Tao Clinic. *Healthcare services provided at these NGO clinics are free-of-charge.*
****Legal status was classified as "unstable" for participants owning documents preventing them from healthcare entitlement and freedom of movement. These documents included Myanmar identification card, or a community card, or a hospital card or commuting card. Legal status was classified as "stable" for participants owning documents allowing them to healthcare entitlement and freedom of movement. These documents included work permit, certificate of identity (CI) card, ten-year resident card, Thai identification card, birth certificate.

Options listed of health seeking behaviour in case of fever persistence originate from Phase I qualitative analysis

Study title: Fever and health-seeking behaviour among migrants living along the Thai-Myanmar border: a mixed-methods study

Table S4. Declared criteria of healthcare service preference in case of fever persistence among Phase II participants.

	Health post (n=73)	Private clinic (n=87)	PCU* (n=30)	NGO Clinics** (n=92)	Hospital (n=68)
**Reasons for attending**					
Access	54 (74.0)	41 (52.9)	22 (73.3)	48 (52.2)	30 (44.1)
Cost	41 (56.2)	2 (2.3)	21 (70.0)	67 (72.8)	10 (14.7)
Quality of care	12 (16.4)	55 (63.2)	2 (6.7)	67 (72.8)	46 (67.7)
Force of habit	42 (57.5)	31 (35.6)	11 (36.7)	45 (48.9)	12 (17.7)
Word of mouth	20 (27.4)	8 (9.2)	3 (10.0)	37 (40.2)	7 (10.3)
Respectful management	32 (43.8)	37 (42.5)	11 (36.7)	65 (70.7)	26 (38.2)

Data are shown in number (%).

*PCU – Primary care unit
**NGO Clinics– Non-governmental organisation, including Shoklo Malaria Research Unit (SMRU) clinics and Mae Tao Clinic
