# Supplementary material for: Fever and health-seeking behaviour among migrants living along the Thai-Myanmar border: a mixed-methods study
Source: BMC Infect Dis. 2023 Jul 31;23:501. doi: 10.1186/s12879-023-08482-8 (PMC10388507; doi:10.1186/s12879-023-08482-8)
Supplement: Supplementary file 4 — Additional file 4. Supplementary study information. The PDF document contains additional information, and insights of the study, which include What is already known? What are the new findings? What do the new findings imply? Strength and Limitations of the study. [file 12879_2023_8482_MOESM4_ESM.pdf]

Supplementary study information.

What is already known?

Migrants on the Thai-Myanmar border suffer from precarious political, economic and social conditions, marked by limited access to healthcare. For migrants with febrile illness, access to healthcare workers with malaria point-of-care rapid test and effective treatment has been vastly extended, but declining malaria has complexified how fever is perceived, what its believed causes are, and therefore what therapeutic actions are undertaken.

What are the new findings?

The concept of fever was found to be complex among migrants on the Thai-Myanmar border, with a broad range of terms, symptoms and believed causes associated to it. Although health services seemed diverse on the Thai-Myanmar border, we report unequal access and provide insights on the determinants influencing migrants in their journey to care. Combining qualitative and quantitative analyses, we were able to demonstrate that distance and legal status were key barriers in access healthcare in this region.

What do the new findings imply?

Despite their contribution to the economic development of the region, we showed that migrants from the Thai-Myanmar border had limited access to the public health system in Thailand, warranting more inclusiveness for febrile patients without a legal status, for a better control of infectious diseases. Regarding the complexity of the journey to care and its determinants, future research should carry out a situational analysis prior to any intervention: currently, most studies assume fever as a unique condition and include participants who may not be representative of

the whole population spectrum. Exclusion of vulnerable populations like migrants may, therefore, undermine infectious diseases prevention and control programmes.

Strength and Limitations of this study

Strengths

- The study team purposively explored into context of vulnerable and marginalized population living along Thai-Myanmar border locating within Tak province, Thailand.
- The data uncovered certain traits and behaviour to healthcare seeking practices when encountering febrile illness and other illnesses.

Limitations:

- The study participants and data collected were not representing the whole population group in the areas of study, due to high mobility and/or casual migrations, and possible lack of access to healthcare services.
- During the period of data collection, there was an outbreak of Chikungunya in data collection areas. Such outbreak may result in limitation of general health seeking behaviour.
